# De novo generation of the NPM-ALK fusion recapitulates the pleiotropic phenotypes of ALK+ ALCL pathogenesis and reveals the ROR2 receptor as target for tumor cells

**DOI:** 10.1186/s12943-022-01520-0

**Published:** 2022-03-04

**Authors:** Loélia Babin, Alice Darchen, Elie Robert, Zakia Aid, Rosalie Borry, Claire Soudais, Marion Piganeau, Anne De Cian, Carine Giovannangeli, Olivia Bawa, Charlotte Rigaud, Jean-Yves Scoazec, Lucile Couronné, Layla Veleanu, Agata Cieslak, Vahid Asnafi, David Sibon, Laurence Lamant, Fabienne Meggetto, Thomas Mercher, Erika Brunet

**Affiliations:** 1 Laboratory of the « Genome Dynamics in the Immune System », Équipe Labellisée La Ligue Contre Le Cancer, Université de Paris, Université Paris Saclay, INSERM UMR 1163, Institut Imagine, Paris, France; 2 Programme PEDIAC, Equipe labellisée Ligue Contre le Cancer, OPALE Carnot Institute, Université Paris Saclay, INSERM Unité U1170, Gustave Roussy Cancer Campus, 114, rue Édouard-Vaillant, 94805 Villejuif, France; 3 Laboratory of Lymphocyte Activation and Susceptibility to EBV infection, Université de Paris, INSERM UMR1163, Institut Imagine, Paris, France; 4 INSERM U1154, CNRS UMR 7196, Sorbonne Universités, Museum National d’Histoire Naturelle, 43 rue Cuvier, F-75231 Paris, France; 5 PETRA platform, AMMICa, University Paris Saclay, CNRS-UMS 3655 Inserm US23, Gustave Roussy, 94805 Villejuif, France; 6 Department of Pediatric and Adolescent Oncology, Gustave Roussy, 94805 Villejuif, France; 7 Department of Pathology, AMMICa CNRS UMS3655 Inserm US23 Université Paris Saclay, Gustave Roussy, 94805 Villejuif, France; 8 Laboratory of Onco Hematology, Hôpital Necker - Enfants Malades, Assistance Publique Hôpitaux de Paris (APHP); Laboratory of Normal and pathological lymphoid differentiation, University of Paris, INSERM U1151, INEM Institute, Paris, France; 9 Université de Paris, Institut Necker-Enfants Malades (INEM), INSERM U1151, and Laboratory of Onco-Hematology, AP-HP Hôpital Necker Enfants-Malades, Paris, France; 10 Université Toulouse III-Paul Sabatier, Laboratoire d’Excellence Toulouse Cancer-TOUCAN, Équipe Labellisée La Ligue Contre Le Cancer, CNRS UMR5071, Inserm, UMR1037, CRCT, F-31000 Toulouse, France

**Keywords:** ALK+ ALCL, Lymphoma, NPM-ALK fusion, CRISPR/Cas9 models, WNT, ROR2, Biomarker, Therapeutic targets

## Abstract

**Background:**

Anaplastic large cell lymphoma positive for ALK (ALK+ ALCL) is a rare type of non-Hodgkin lymphoma. This lymphoma is caused by chromosomal translocations involving the anaplastic lymphoma kinase gene (ALK). In this study, we aimed to identify mechanisms of transformation and therapeutic targets by generating a model of ALK+ ALCL lymphomagenesis ab initio with the specific NPM-ALK fusion.

**Methods:**

We performed CRISPR/Cas9-mediated genome editing of the NPM-ALK chromosomal translocation in primary human activated T lymphocytes.

**Results:**

Both CD4+ and CD8+ NPM-ALK-edited T lymphocytes showed rapid and reproducible competitive advantage in culture and led to in vivo disease development with nodal and extra-nodal features. Murine tumors displayed the phenotypic diversity observed in ALK+ ALCL patients, including CD4+ and CD8+ lymphomas. Assessment of transcriptome data from models and patients revealed global activation of the WNT signaling pathway, including both canonical and non-canonical pathways, during ALK+ ALCL lymphomagenesis. Specifically, we found that the WNT signaling cell surface receptor ROR2 represented a robust and genuine marker of all ALK+ ALCL patient tumor samples.

**Conclusions:**

In this study, ab initio modeling of the ALK+ ALCL chromosomal translocation in mature T lymphocytes enabled the identification of new therapeutic targets. As ROR2 targeting approaches for other cancers are under development (including lung and ovarian tumors), our findings suggest that ALK+ ALCL cases with resistance to current therapies may also benefit from ROR2 targeting strategies.

**Supplementary Information:**

The online version contains supplementary material available at 10.1186/s12943-022-01520-0.

## Background

Anaplastic large cell lymphoma with anaplastic lymphoma kinase gene translocations (ALK+ ALCL) is a mature T-cell lymphoma that primarily affects lymph nodes, but also gives rise to tumors in extranodal organs and occasional characteristic skin lesions. Although the majority of patients recover with first line chemotherapy, 10 to 30% of patients relapse with a poor prognosis [1–4].

In terms of immune phenotypes, ALK+ ALCL tumor cells are primarily characterized by constant CD30 antigen expression in addition to a variety of other immunologic phenotypes (summarized in [5]). While the majority of ALK+ ALCL tumors are CD4+ (approx. 70%), approximately 10% of patient samples exhibit a CD8+ phenotype. Null T-cell phenotypes and rarer CD4 + CD8+ phenotypes have also been described. Only 4% of ALK+ ALCL patient cells express T-cell receptors (TCR) at the cell surface, whereas rearrangements at the genomic TCR locus have often been observed (> 75%) [6]. The majority of ALK+ ALCL tumor cells have been shown to express at least one T-cell specific marker. This pleiotropic combination of markers observed in ALK+ ALCL has rendered the identification of the cell of origin difficult, with reports proposing either a thymic or a peripheral origin [6–8].

Between 60 and 80% of ALK+ ALCL cases harbor the NPM-ALK chromosomal translocation, which leads to constitutive activation of the ALK protein. Uncontrolled activation of ALK induces several cascades of signaling pathways, including phosphorylation of STAT3, which is important for the maintenance of the malignant phenotype [9–11].

The oncogenic potential of the *NPM-ALK* fusion gene was first demonstrated in vitro using murine cell lines and primary cells. Several in vivo approaches in mice have failed to phenocopy human ALK+ ALCL and rather often yield B-cell lymphomas [12–16]. More recently, transduction of primary human CD4+ T lymphocytes with the *NPM-ALK* transgene has led to in vitro transformation of the cells [7, 8, 17] and in vivo tumor formation [7, 17]. However, lentiviral expression does not precisely reproduce gene dosage nor the spatiotemporal variations in gene expression associated with the various stages of differentiation. Moreover, these models lack other potentially important oncogenic features resulting from chromosomal translocation formation, including the reciprocal fusion gene, potential haplo-insufficiency of NPM1 or chromatin states resulting from the repositioning of translocated chromosomes. As high expression levels of NPM-ALK is toxic [18] and a limited number of clones are able to grow despite high transduction efficiency, it is likely that prolonged selection is required to establish the appropriate conditions in these models. Interestingly, transplantation of CRISPR/Cas9-edited cells from murine fetal liver hematopoietic stem cells into mice led to the development of CD30+ T-cell lymphomas with spleen and secondary organ involvement. However, no nodal tumors were detected, and cells were primarily CD3+ and CD4 + CD8+ (uncommon immunophenotypes found in humans).

In the present study, we demonstrate high-efficacy transformation of primary human activated T lymphocytes upon CRISPR/Cas9-mediated engineering of the NPM-ALK translocation. Translocated human T lymphocytes increased survival for over three months and presented with various phenotypes. Murine recipients transplanted with the human NPM-ALK lymphocytes developed systemic disease within a median of three months, with nodal, extranodal and sporadic skin involvement, thus resembling the disease observed in humans. Interestingly, we found that tumor formation recapitulated the immune phenotypic diversity (including the less common CD8+ tumors), the histological pattern (large and small tumor cells) and the transcriptomic signature of ALK+ ALCL patient cells. Transcriptomic analysis using this oncogenic model revealed specific activation of the WNT signaling pathways in ALK+ ALCL, which was further confirmed in patient data. Furthermore, gene analysis of the progression from wild type lymphocytes into tumors revealed that the ROR2 transmembrane receptor was upregulated during oncogenesis. As the ROR2 transmembrane receptor is exclusively expressed in embryonic tissue, it represents a promising surface target to treat ALK+ ALCL patients.

Overall, we have established a unique efficient genome-editing strategy to model gene fusions in primary human lymphocytes and characterized the steps of normal T-cell progression into transformed ALK+ lymphomas in vivo. The ROR2 cell surface receptor represents a diagnostic alternative and potential therapeutic target for ALCL patients with resistance to chemotherapy.

## Methods

### Primary cells and cell lines

PBMCs were isolated using SepMate™-50 (IVD) (StemCell Technologies #85450) following manufacturer’s instructions. PBMCs were activated 5 to 7 days on coated plates with anti-CD3-OKT3 (Biolegend #317325 RRID: AB_11147370) and 1 ng/uL anti-CD28 (eBioscience #16–0289-81 RRID: AB_468926) in RPMI medium (Invitrogen) supplemented with 20% heat-inactivated Fetal Bovin Serum (GIBCO). After activation cells were directly transfected with the RiboNucleoProtein RNP/Cas9 complex. The patient-derived xenograft (PDX) model was obtained from a tumor biopsy of a patient with newly diagnosed ALK+ ALCL and cells were engraved subcutaneously in NSG mice by the team of Dr. D. Sibon. ALK+ ALCL cell lines and patient-derived xenograft (PDX) were cultivated in RPMI (Invitrogen) medium supplemented with 20% heat inactivated Fetal Bovine Serum (GIBCO).

### CRISPR/Cas9 transfection and translocation frequency

T lymphocytes were transfected at 5 to 7 days post CD3/CD28 activation, with the RNP/Cas9 complex using the 4D Nucleofector Amaxa technology (Lonza) (using the gRNA^NPM^ and gRNA^ALK^ and the Cas9 protein (quantity ratio 2:1) and as described in [19]). IL-2 (40 U/mL) was added in the media once at the time of transfection but never used afterwards. Transfected cells were long term maintained in 20% heat-inactivated FBS complemented RPMI medium. gRNA sequences are listed in Supplementary Materials.

### TCR analysis

TCRγ analysis was performed as previously described [20].

### PCR-based translocation detection and frequency

Serial dilutions of DNA from transfected cells enable the assessment of translocation frequency as previously described in Supplementary Reference [21]. Primer sequences are listed in Supplementary Materials.

### NGS breakpoint junction sequencing

The first-round of PCR was performed using primers containing adapter sequences. The second round of PCR was performed using primers containing barcode sequences. PCR products were sequenced using 2 × 100 cycles (paired-end reads, 100 nucleotides) on the Illumina NovaSeq6000 instrument (Illumina). Sequences were analyzed using previously described software to identify indels and microhomology [22] and we analyzed junction sequences with at least 2 reads. Primer sequences are listed in Supplementary Materials.

### RNA-seq and bioinformatics analysis

Sequencing was carried out using 2 × 100 cycles (paired-end reads, 100 nucleotides) for all samples on the Illumina NovaSeq6000 instrument. Reads were quantified with salmon v0.14.1 (genome GRCh38) and differential analysis was performed using the R package DESeq2 (R version 4.0.3 and DESeq2 version 1.30.1). No statistical methods were used to predetermine sample size. RNAseq experiments were performed in triplicates. All GSEA analyses (version 4.1.0) were performed using the pre-ranked mode because of the weak number of samples for each condition in data coming from the model. In order to identify genes harboring a strong progressive up-regulation in the model’s dataset, genes that harbored an overexpression associated to a LFC2 higher than 2 (and obligatorily associated with an adjusted *p*-value lower than 5%) both between conditions WT (wild type) and NPM-ALK in vitro as well as between conditions NPM-ALK in vitro and NPM-ALK in vivo were selected.

### Animal experiments

NSG immunodeficient mice (NOD.Cg-Prkdc(scid) Il2rg(tm1Wjl)/SzJ (the Jackson Laboratory, Bar Harbor, ME, USA) were maintained at the Gustave Roussy preclinical facility and NOD/SCID Gamma (NSG NOD-prkdcscid) mice (Janvier Labs) for subcutaneous experiments were housed at the CRCT facility. For xenograft tumor assay, a total of 3 × 10^6^ ALKIma1 cells were injected subcutaneously into both flanks of 5-week-old female NSG mice as described [7]. For intravenous injections, 8 to 12-weeks old NSG mice were irradiated at 1.5 Gy, and 0.7 to 3 million human cells were injected intravenously (i.v.). Disease progression was monitored by flow cytometry of mouse peripheral blood drawn periodically by submandibular bleeds. Mice were sacrificed when engraftment reached at least 30% or upon reaching a defined disease endpoint.

### Histological analysis

Subcutaneous tumors or organs were excised and sections were fixed in 10% neutral buffered formalin and embedded in paraffin for staining with H&E. For histological analyses, sample organs were stained with hematoxylin and eosin. Briefly, the slides were heat-treated for antigen retrieval using CC1 buffer (pH 8) and incubated with pre-diluted primary antibodies to anti-ALK1 (clone ALK-01), anti-CD30 (clone Ber-H2), anti-CD4 (clone SP35) and anti-CD3 (clone 2GV6) (all from Ventana, Roche Diagnostics) and anti-ROR2 (Abcam #ab218105). Epitopes were subsequently visualized using the OptiView DAB detection method (Ventana, Roche Diagnostics) and nuclei were counterstained with haematoxylin. For interpretation, the slides were evaluated by light microscopy.

Additional materials and methods are described in the Supplemental Material.

## Results

### Generation of t(2;5)(p23;q35) in human primary T lymphocytes induces efficient growth and proliferation of cells through endogenous NPM-ALK activation

**Fig. 1:**
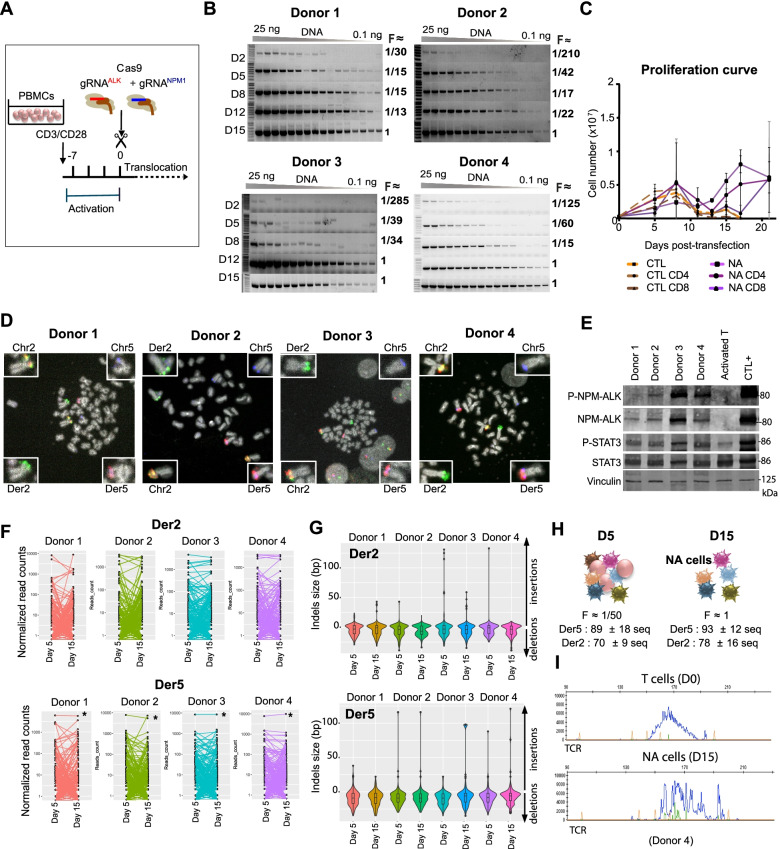
Generation of NA cells derived from activated T lymphocytes. **A.** CRISPR/Cas9-based strategy performed to obtain ALK+ ALCL model cells from activated T lymphocytes. PBMCs were collected from healthy donor blood and activated with anti-CD3/CD28 for 7 days. Activated cells were transfected with RNP complex (Cas9 + gRNA^*NPM1*^/gRNA^*ALK*^). DNA was extracted at day 0 and then every three days to estimate translocation frequency overtime. **B.** Translocation frequency assessed by PCR amplification of derivative chromosome 5, Der5, from 2 (D2) to 15 days (D15) post-transfection (*n* = 4 independent donors). PCR were performed in duplicate on DNA dilutions (dilutions: 25 ng to 0.1 ng). The translocation frequency (F) was calculated as described in [21] presuming that a human diploid cell contains approximately 6 pg of DNA. **C.** Proliferation curve of control T lymphocytes (control cell groups (CTL): unsorted, CD4 + CD8-, CD8 + CD4-) and CRISPR/Cas9-transfected lymphocytes (NA cell groups: unsorted, CD4 + CD8-, CD8 + CD4-). Median with standard deviation for *n* = 3. **D.** Representative image of metaphases (FISH analysis) obtained from NA cells, 1 month post-transfection. Break-apart probe (green + red): *ALK* gene; blue probe: *NPM1* gene. **E.** Western blot analysis of NPM-ALK, STAT3, phosphorylated NPM-ALK (P-NPM-ALK) and phosphorylated STAT3 (P-STAT3) in NA cells from Donors 1 to 4, at 1 month post-transfection. Activated T lymphocytes were used as negative controls, and an ALK+ ALCL cell line was used as a positive control (CTL+). Vinculin was used as the loading control. **F.** Evolution of sequencing read numbers for each type of translocation breakpoint junction sequence obtained by targeted sequencing (for der2 and der5) at day 5 and day 15 after transfection. Each curve represents the number of normalized reads of one single sequence. For Der5, one sequence is overrepresented (*) (corresponding to a ΔAG deletion). **G.** Violin plots of indel size (in bp for deletions and insertions) for Der2 and Der5 breakpoint junction sequences, at day 5 and day 15 for the four donors. **H.** Schematic diagram of NA cell selection showing the number of breakpoint junctions at day 5 and 15 post-transfection (normalized mean ± SD of number of different junction sequences observed per transfection and calculated from the sequencing of at least 900 reads). F = translocation frequency estimated by PCR of DNA dilutions (see Fig. 1B). **I.** Analysis of TCRγ clonality via multiplex PCR of activated T lymphocytes (day of transfection: D0) and NA cells 15 days (D15) post-transfection (corresponding to Donor 4)

To recapitulate the t(2;5)(p23;q35) translocation in situ*,* CD3 and CD28 activated T lymphocytes isolated from peripheral blood mononuclear cells (PBMCs) from healthy donors were transfected with the ribonucleic protein (RNP)/Cas9 complexes with gRNA^*NPM1*^ (targeting the *NPM1* gene) and gRNA^*ALK*^ (targeting the *ALK* gene) (Fig. 1A**)**. Transfected cells were maintained in IL-2-free medium for long-term culture. A total of 15 independent donors were used in this study (Supplementary Table S1). This strategy led to efficient generation of *NPM-ALK* genomic fusions (translocation frequency > 1.7% at day 5 post-transfection) (Fig. 1B)**.** In control lymphocytes, we observed rapid cell proliferation arrest in culture medium without addition of IL-2 as previously reported [23] (Fig. 1C and Supplementary Fig. S1A). By contrast, CD4+ and CD8+ activated T lymphocytes treated with gRNA^*NPM1*^ and gRNA^*ALK*^ started to proliferate 12 to 15 days after transfection (Fig. 1C and Supplementary Fig. S1A). Accordingly, translocation frequency rapidly increased to 100%, thereby demonstrating positive selection of cells carrying the t(2;5)(p23;q35) translocation (hereafter referred to as “NA cells”) (Fig. 1B). We obtained a similar result using a different pair of gRNAs, thus indicating that cell proliferation and positive selection were due to the translocation (Supplementary Fig. S1B). We confirmed the formation of the t(2;5)(p23;q35) chromosomal translocation in every NA cell by fluorescence in situ hybridization (FISH) analysis (Fig. 1D). NA cells expressed a functionally active form of NPM-ALK (constitutive ALK and STAT3 phosphorylation) (Fig. 1E**)**.

We further confirmed the efficacy of our approach by sequencing the breakpoint junctions from the pool of NA cells. Strikingly, approximately 100 distinct fusion sequences were amplified at day 5, which were still detected at day 15 post-transfection, thereby demonstrating the generation of highly polyclonal cultures (Fig. 1F and Supplementary Table S2). The breakpoint junctions showed small deletions/insertions (median size: − 4 bp for Der2 and + 4 bp for Der5) with few microhomologies (median size of microhomology: < 2 bp, Fig. 1G and Supplementary Table S2), typical of the classical non-homologous end joining repair pathway, the predominant translocation mechanism in human cells after double-strand breaks [24]. TCR analysis of DNA from NA cells at day 15 showed multiple TCR peaks and, together with the variety of breakpoint junctions detected, ascertained the polyclonal nature of the edited NA lymphocytes (Fig. 1F to I). NA cells from the four donors were maintained in culture medium without IL-2 for 3 months. At this time point, 1 to 2 different translocation breakpoint junctions were detected, indicating an expected selection of a few clones during culture (Supplementary Fig. S1C). Furthermore, 13 experiments from independent donors led to positive selection and expansion of NA cells for at least 1.5 months (summarized in Supplementary Table S1), while viable control cells were not detected after 18 days. Overall, the results demonstrate that this strategy leads to quick, reproducible and efficient CRISPR/Cas9-mediated engineering of the t(2;5)(p23;q35) in pre-activated human primary T lymphocytes.

### A t(2;5)(p23;q35) engineered cell line (ALKIma1) represents a novel model of ALK+ ALCL

**Fig. 2:**
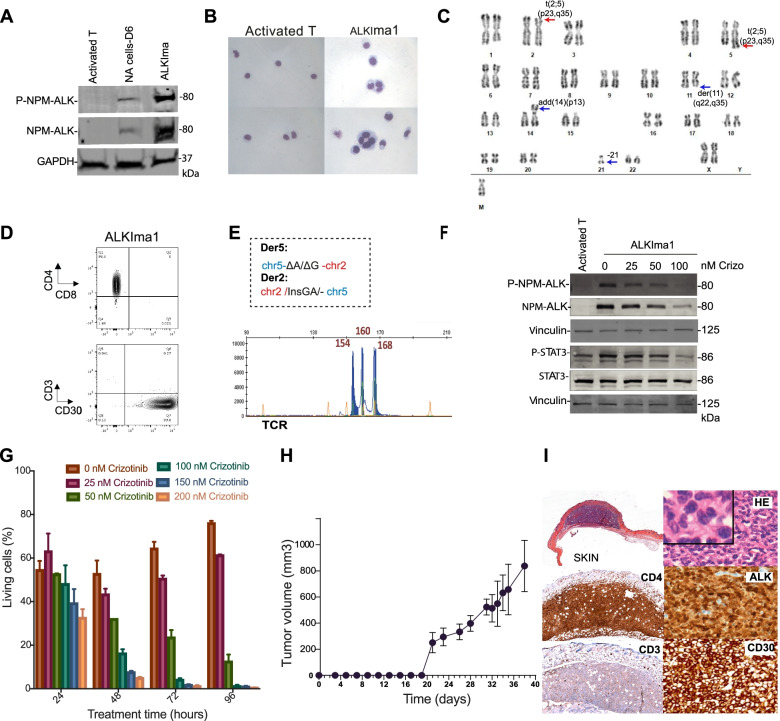
In vivo tumors derived from the ALKIma1 cell line. **A.** Western blot analysis of NPM-ALK, and phosphorylated NPM-ALK (P-NPM-ALK) in CD4+ NA cells, 6 days post-transfection (NA cells-D6) and the ALKIma1 cell line. GAPDH was used as a loading control. **B.** Cytospin analysis of primary activated T cells (5 days post-activation) and ALKIma1 cells. **C.** Karyotype analysis of ALKIma1 cells. Red arrows show the t(2;5)(p23;q35) translocation, blue arrows indicate additional chromosomal events. **D.** Flow cytometry assessment of CD4, CD8 and CD3, CD30 expression levels in ALKIma1 cells. **E.** Der2 and Der5 sequences obtained by Sanger sequencing of ALKIma1 cells, and analysis of TCRγ clonality via multiplex PCR of ALKIma1 cells. **F.** Western blot analysis of NPM-ALK, STAT3, phosphorylated NPM-ALK (P-NPM-ALK) and phosphorylated STAT3 (P-STAT3) in ALKIma1 cells treated with crizotinib for 48 h versus untreated ALKIma1 cells. Activated T lymphocytes were used as a negative control. Vinculin was used as a loading control. Crizo: crizotinib. **G.** Cell survival analysis of ALKIma1 cells treated with increasing doses of crizotinib for 24, 48, 72 and 96 h. **H.** Temporal evolution of tumor volume in mice injected with ALKIma1 cells (subcutaneous injection). **I.** Histologic analysis of ALKIma1 tumor cells in skin nodules: H&E staining, anti-ALK, anti-CD3, anti-CD4 and anti-CD30

We next transfected purified CD4+ lymphocytes, which represent the most typical tumor phenotype in ALK+ ALCL, and were able to express active NPM-ALK protein as early as 6 days post-transfection (Fig. 2A). We established a stable cell line from Donor #11 (hereafter referred to as ALKIma1 cells, Supplementary Table S1). ALKIma1 cells grew for more than 100 days, presented with increased phosphorylation of NPM-ALK (Fig. 2A), typical ALCL morphology with large cells and a large nucleus (Fig. 2B) and showed robust telomerase activity, in agreement with the immortalized phenotype (Supplementary Fig. S1D).

Cytogenetic analysis of ALKIma1 cells revealed a nearly diploid karyotype with the typical reciprocal t(2;5)(p23;q35) translocation (Fig. 2C). After 4 months in culture, ALKIma1 cells, which are CD4+ and CD3-, displayed high levels of CD30 (Fig. 2D**)**. These cells were clonal with a single sequence for each translocation breakpoint and a clonal TCR (Fig. 2E). Importantly, ALKIma1 cells expressed functional p53 with G1/S checkpoint activation (Supplementary Fig. S1E, F) as found in most ALK+ ALCL patient tumors (p53 is mutated in < 10% of cases) [25]. We also showed functional activation of the downstream STAT3 pathway (Fig. 2F) and inhibition of NPM-ALK phosphorylation leading to cell death upon treatment with crizotinib, a specific ALK inhibitor used in the clinic (Fig. 2F, G) [26].

To evaluate whether the ALKima1 cell line gives rise to tumors in vivo, we performed subcutaneous xenotransplantation in immunodeficient NSG mice (Fig. 2H). Rapid tumor growth was observed in recipient animals (6 tumors, 3 mice). All mice exhibited skin nodules without dermis and subcutis hyperplasia (Supplementary Fig. S1G). Histological analysis of the subcutaneous tumors showed ALK+ ALCL cell morphology (primarily common type cells with a group of small cells). Immunohistochemistry (IHC) experiments confirmed robust nuclear and cytoplasmic expression of ALK typical of the NPM-ALK translocation and CD30 expression (Fig. 2I). The cells were CD4+, primarily CD3-, CD2+, CD5+, CD7+, CD20-, GranzymeB- and Perforin+, a representative phenotype of ALK+ ALCL tumors (Fig. 2I and Supplementary Fig. S1H). These results show that the CRISPR/Cas9-generated ALKIma1 cell line represents a novel and bona fide cellular model of ALK+ ALCL tumors.

### Induction of t(2;5)(p23;q35) recapitulates the phenotypic diversity observed in ALK+ ALCL patients

**Fig. 3:**
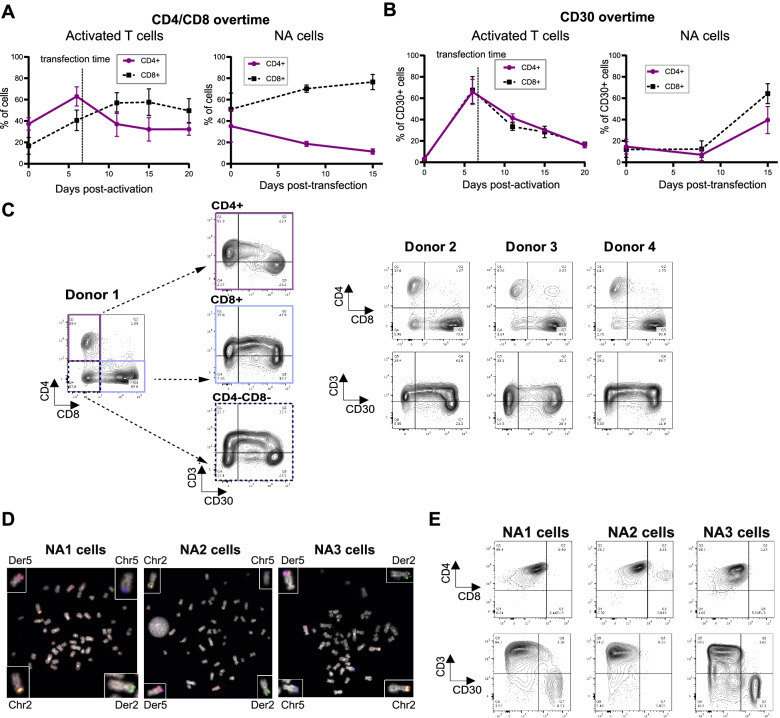
Immunophenotype of NA cells. **A.** CD4+ and CD8+ cell survival overtime (analyzed by flow cytometry) for primary activated T lymphocytes (between 0 and 20 days post-activation) and NA cells (between 0 and 15 days post-transfection). The time of transfection is indicated by a dashed line for primary activated T lymphocytes (*n* = 3). **B.** CD30 expression for each cell population (CD4+ or CD8+ cells) for primary activated T lymphocytes (between 0 and 20 days post-activation) and NA cells (between 0 and 15 days post-transfection). The transfection timepoint is indicated by a vertical dashed line for the primary activated T lymphocytes (*n* = 3). **C.** Flow cytometry assessment of the CD4, CD8, CD3 and CD30 markers. For all donors, cell populations were obtained at day 15 post-transfection. Subpopulations (CD4 + CD8-, CD8 + CD4- and CD4-CD8-) were also analyzed for Donor 1 (left). **D.** Representative image of metaphases (FISH analysis) of NA1, NA2 and NA3 cells, 1 month post-transfection. Break-apart probe (green + red): *ALK* gene; blue probe: *NPM1* gene. **E.** Flow cytometry assessment of CD4, CD8, CD3 and CD30 expression in NA1, NA2, NA3 cells (1 month post-transfection, time point corresponding to mice injections)

ALK+ ALCL patients are characterized by various immune phenotypes. Most tumors are CD4+, although tumors can also exhibit a CD8+ phenotype (10%), a null T-cell or a CD4 + CD8+ phenotype (rare). During in vitro expansion of mixed CD4+ and CD8+ T lymphocyte populations, CD8+ cells were selected overtime (Fig. 3A and Supplementary Fig. S2A). NA cells obtained after editing of unsorted CD4+ and CD8+ activated lymphocytes showed a similar bias toward CD8+ NA cell selection (Fig. 3A, Supplementary Fig. S2B). Interestingly, both CD4+ and CD8+ NA cells showed a similar increased expression of the marker CD30 at 10 days post-transfection, while wild type lymphocytes displayed decreased CD30 expression levels (Fig. 3B). We also observed a CD3- and CD30+ specific cell population reminiscent of typical ALK+ ALCL cells (Fig. 3C). Furthermore, we detected a CD4-CD8- cell population mostly comprised of CD30+ cells (Fig. 3C), which is a phenotype also found in primary ALCL tumors [27]. According to our results, editing of primary human T lymphocytes led to a variety of immune phenotypes, as found in ALK+ ALCL patients. After CD4+ cell sorting and transfection with gRNA^*NPM1*^ and gRNA^*ALK*^*,* we also obtained a collection of CD4+ NA cells from independent experiments (e.g., NA1, NA2 and NA3 cells, Fig. 3D, E and Supplementary Fig. S3A). Overall, we were able to recapitulate in vitro most of the different immune phenotypes found in ALK+ ALCL.

### Early polyclonal NPM-ALK-translocated T lymphocytes induce in vivo disease development with diverse phenotypes, including CD8+ tumors

**Fig. 4:**
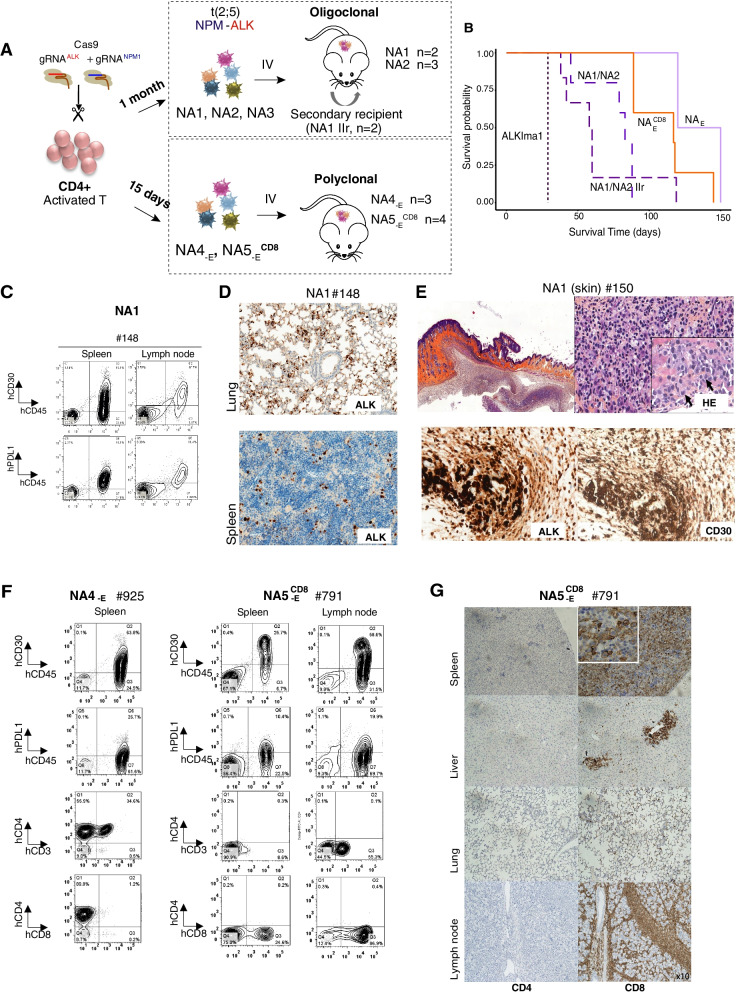
In vivo ALK+ ALCL lymphomagenesis derived from NA cells. **A.** Schematic illustration of the experiment: NA1, NA2 and NA3 cells were intravenously injected into NSG mice at 1 month post-transfection. Tumor cells from NA1 cells were injected in secondary recipients (NA1 IIr). In parallel, NA4_-E_ (unsorted cells) and NA5_-E_^CD8^ (CD8+) cells were intravenously injected in NSG mice at 15 days post-transfection. IV: intravenous injection. **B.** Kaplan-Meier survival curve of first and second recipient mice intravenously injected with different pools of NA cells obtained from various time points after transfection (15 days (NA4_-E,_ NA5_-E_^CD8^), 1 month (NA1 and NA2) or ALKIma1 cell line). IIr: secondary mouse recipients. **C.** Flow cytometry assessment of CD45, CD30 and PDL1 expression in tumors derived from NA1 cells (spleen and lymph node) for mouse #148. **D.** Histologic analysis of tumors derived from NA1 cells: anti-ALK in lung and spleen tumors for mouse #148. **E.** Histologic analysis of tumors from NA1 cells in skin nodules: H&E staining, anti-CD30, anti-ALK for mouse #150. **F.** Flow cytometry assessment of CD45, CD30, PDL1, CD4, CD8 and CD3 expression in tumor cells (spleen and lymph node) from mice #925 (injected with NA4_-E_) and mouse #791 (injected with NA5_-E_^CD8^ cells). **G.** Histologic analysis of tumors in spleen, liver, lung and lymph node from mouse #791 injected with NA5_-E_^CD8^ cells: anti-CD4 and anti-CD8

To avoid possible clonal selection bias upon long-term in vitro culture, we injected NSG recipient mice with NA cells early after transfection, when engineered NA cells were polyclonal with heterogeneous expression of the various markers. We performed intravenous injections into mice to assess the capacity of our model to yield disseminated disease in vivo (Fig. 4A). First, we injected the NA1, NA2 and NA3 cells into mice after 1 month in culture (Fig. 3D, E). At the time of injection, the NA1 and NA3 cells were CD4+ with a small population of CD30+ cells, while the NA2 cells were CD30- with a small population of CD4 + CD8+ cells (Fig. 3E). CD45+ human cells were detected in the blood samples as soon as 6 weeks post-injection, with the exception of the NA3 cells (Supplementary Fig. S3B). In terms of latency, mice injected with NA1 and NA2 cells (primary and secondary recipients) developed tumors that reached ethical endpoint criteria within 86–88 days (Fig. 4B). The NA3 cells did not cause disease in any recipient mouse (7/7) during the follow-up at 14 months (Supplementary Table S1). Interestingly, the mice injected with NA1 and NA2 cells developed disseminated disease in lymph nodes and extranodal organs (liver, spleen and lungs) **(**Fig. 4C, D**,** Supplementary Fig. S3C to E). We also observed skin involvement as reported in ALK+ ALCL patients (Fig. 4E and Supplementary Fig. S4A). Tumor cells isolated from the various organs were primarily CD3- but expressed ALK (high expression levels; both nuclear and cytoplasmic), CD30 and PDL1 (Fig. 4C, E**,** and Supplementary Fig. S3D, E). Interestingly, the NA2 cells [initially CD30- **(**Fig. 3E)] also yielded tumors with high levels of CD30 expression (Supplementary Fig. S3E**)**. Tumor cells from NA1 cells that were reinjected into secondary recipients led to tumor development in lymphoid organs (Fig. 4A, B and Supplementary Fig. S3F).

Morphologically, mice developed tumors and skin nodules with a dense infiltrate of both small and large cells, which is referred to as mixed pattern in human pathology. Large cells had the attributes of so-called “hallmark” ALCL cells with a kidney-shaped nucleus and abundant cytoplasm. Small cells exhibited an irregular chromatic nucleus centrally located within a pale cytoplasm, referred to as “fried egg” cells (example of H&E staining in Fig. 4E and Supplementary Fig. S4A). Histological analysis confirmed high expression levels of the CD30 marker (Fig. 4E**,** Supplementary Fig. S4A). The NA1 and NA2 cells were oligoclonal at the time of injection, as shown by TCR analysis (Supplementary Fig. S4B). Unexpectedly, the NA2 cells led to CD4 + CD8+ tumor formation in one of the mice (Supplementary Fig. S4A), which probably arose from the few double positive cells present at the time of injection (Fig. 3E).

We then injected NA cells as early as 15 days after transfection, when about 100 clones were detected (Fig. 1F) (hereafter referred to as NA_-E_ cells, “-E” for early, Fig. 4A). For this experiment, we injected either a mixed population of CD4+ cells and CD8+ cells (NA4_-E_) or a pure population of CD8+ cells (NA5_-E_^CD8^) (Supplementary Fig. S5A). High translocation frequency was noted at the time of injection (Supplementary Fig. S5B). We injected 0.7 million cells for each condition (Donors 14 and 15, *n* = 3 and *n* = 4 recipient mice, respectively, Supplementary Table S1). At 6–8 weeks post injection, human CD45+ cells were detectable in the blood of 2/3 NA4_-E_ and all four NA5_-E_^CD8^ recipient mice (Supplementary Fig. S5C). All CD45+ mice developed disseminated disease; the mice reached the ethical endpoint criteria within 118 days (Fig. 4B, F, G, Supplementary Fig. S5D). One mouse injected with NA4_-E_ cells (#925, Fig. 4F and Supplementary Fig. S6A) developed exclusively a CD4+ phenotype, while the other mouse (#923, Supplementary Fig. S5D and S6A) exhibited a mixed phenotype in the spleen (with CD8 + CD4- and CD8-CD4+ cell infiltration) and pure CD8 + CD4- cell infiltration in the lymph nodes (Supplementary Fig. S5D). Notably, DNA extracted from the spleen from this latter mouse revealed two different fusion points, thus indicating engraftment of independent clones (Supplementary Fig. S6B). All four mice injected with NA5_-E_^CD8^ cells developed CD8 + CD4- tumors with either CD3- cells or CD3+ cells, as observed in CD8+ ALCL tumor cells [28] (Fig. 4F, G, Supplementary Fig. S5D). This result indicates that NA cells can yield de novo CD8+ tumors as found in 10% of patients, which constitutes a unique model of CD8+ ALK+ ALCL tumorigenesis.

### Expression profiles from NPM-ALK translocated models reveal activation of canonical and non-canonical WNT pathways in ALK+ ALCL patient samples

**Fig. 5:**
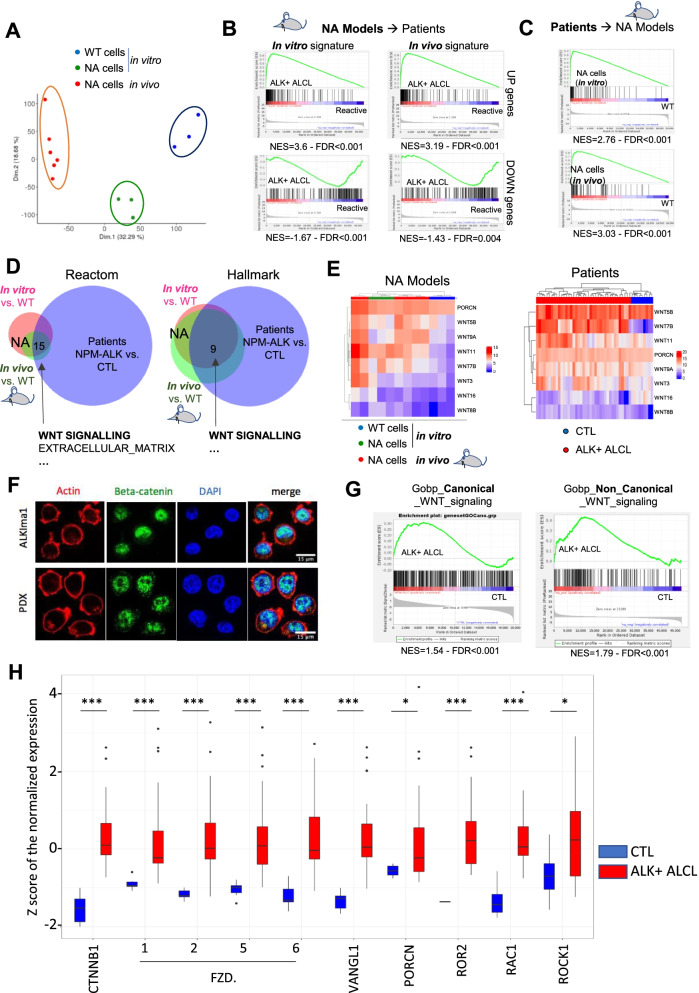
Expression analysis of NA in vitro and in vivo models reveals *WNT* pathway activation in ALK+ ALCL patient cells. **A.** Principal component analysis of the RNAseq data (Dim 1 and 2). Data were scaled to unit variance before performing the representation. **B.** Gene set enrichment analysis of the 200 most differentially upregulated or downregulated genes obtained from the models [comparison of NPM-ALK-edited in vitro models vs. wild type cells (in vitro) or NPM-ALK in vivo models vs. wild type cells] on expression data from ALK+ ALCL patients vs. non tumoral reactive lymph nodes (CTL). UP genes: upregulated genes, DOWN genes: downregulated genes, WT: wild type. **C.** Gene set enrichment analysis using an ALK+ ALCL patient signature of 200 upregulated genes, on expression data from our models: NPM-ALK in vitro vs. wild type cells (upper panel), NPM-ALK in vivo models vs. wild type cells (lower panel). WT: wild type. **D.** Gene set enrichment analysis was performed on the Reactom and Hallmark gene lists for the following pairwise comparisons: NPM-ALK in vitro vs. wild type cells, NPM-ALK in vivo models vs. wild type cells, and NPM-ALK+ patients and non tumoral reactive lymph nodes (CTL). The overlap of significantly enriched genes is represented as Venn diagrams. WT: wild type. **E.** Heatmap representation of expression levels of the enriched WNT genes in the model (left panel) and the patient (right panel) datasets. **F.** Immunofluorescence analysis of beta-catenin in ALKIma1 and PDX cells. Blue: DAPI, green: beta-catenin, red: actin. **G.** Gene set enrichment analysis of the canonical and the non-canonical WNT signaling pathway gene lists to compare ALK+ ALCL patients vs. non-tumoral reactive lymph node expression data (CTL). **H.** Significantly upregulated WNT pathway genes in ALK+ ALCL patient cells compared with reactive lymph nodes (CTL), represented as Z scores computed from Congras et al., 2020 (Journal of Clinical Investigation) using DESeq2. *p*-values were adjusted for multiple comparisons with the Benjamini-Hochberg correction (*: FDR < 0.05, **: FDR < 0.01, ***: FDR < 0.001)

To gain insight into the molecular mechanism of NPM-ALK-mediated transformation, we analyzed the transcriptomes at an early in vitro stage and in in vivo models. We performed RNAseq analysis of three groups: 1-in vitro wild type CD4+ activated lymphocytes [*n* = 3], 2-in vitro NPM-ALK-engineered CD4+ lymphocytes [*n* = 3] and 3-in vivo-derived CD4+ lymphoma cells purified by flow cytometry to isolate CD3+ [*n* = 3] and CD3- populations [*n* = 3]. Principal component analysis clustering showed that the three groups could be clearly segregated (Fig. 5A). Principal component analysis focused on CD3+ and CD3- tumor cells could discriminate CD3+ cells from CD3- cells but mostly on the second axis (Supplementary Fig. S7A, Supplementary Tables S3 and S4) and CD3- cells showed enrichment primarily in cell cycle gene sets (Supplementary Fig. S7B). Based on these minor differences between CD3- and CD3+ cells, gene signatures from in vivo models were later assessed by combining CD3- and CD3+ samples. Using differential gene lists, we determined whether the molecular basis of the models resembled that of ALK+ ALCL patients by performing reciprocal enrichment analyses using transcriptome data from our models and published ALK+ ALCL patient data [7]. First, we assessed upregulated or downregulated gene signatures obtained from our models (gene lists of the 200 most deregulated genes) via gene set enrichment analysis (GSEA) on transcriptomes from ALK+ ALCL patients compared with reactive lymph nodes (controls). Consistently, both in vitro and in vivo upregulated gene signatures showed significant enrichment in the ALK+ ALCL patient samples, and both downregulated gene signatures showed enrichment in the reactive lymph node samples (Fig. 5B). Furthermore, the 200 most upregulated genes in the ALK+ ALCL patient samples (compared with reactive lymph nodes) were also enriched in vitro and in vivo in NA cells (Fig. 5C).

To identify commonly deregulated pathways in in vitro cells, in vivo models and patient samples, we intersected the significantly enriched Reactom and Hallmark gene lists for each of the three groups compared with the respective controls (Fig. 5D**,** Supplementary Tables S5 to S8). The WNT signaling pathway and WNT genes showed consistent enrichment in both NA models and patients (Fig. 5D, E). Among the “extracellular matrix” gene list (Fig. 5D)**,** 29 genes primarily encoding collagens and metallopeptidases were commonly enriched in the three groups (Supplementary Fig. S7C and Supplementary Table S9). The ALKIma1 cells and a PDX model displayed β-CATENIN translocated in the nucleus, indicating functional activation of the canonical WNT pathway **(**Fig. 5F**)**. We found that canonical and non-canonical WNT pathway gene sets were enriched in NA cells and patient cells compared with controls (Fig. 5G and Supplementary Fig. S7D), including canonical (e.g. CTNNB1, FZDs) and non-canonical (e.g. ROR2, RAC1) WNT pathway genes (Fig. 5H). Overall, transcriptomes from our models ascertained that they comparably reproduced molecular characteristics of ALK+ ALCL patient cells and displayed global upregulation of the WNT pathways.

### ROR2 is a robust marker of ALK+ tumor cells in ALK+ ALCL patients

**Fig. 6:**
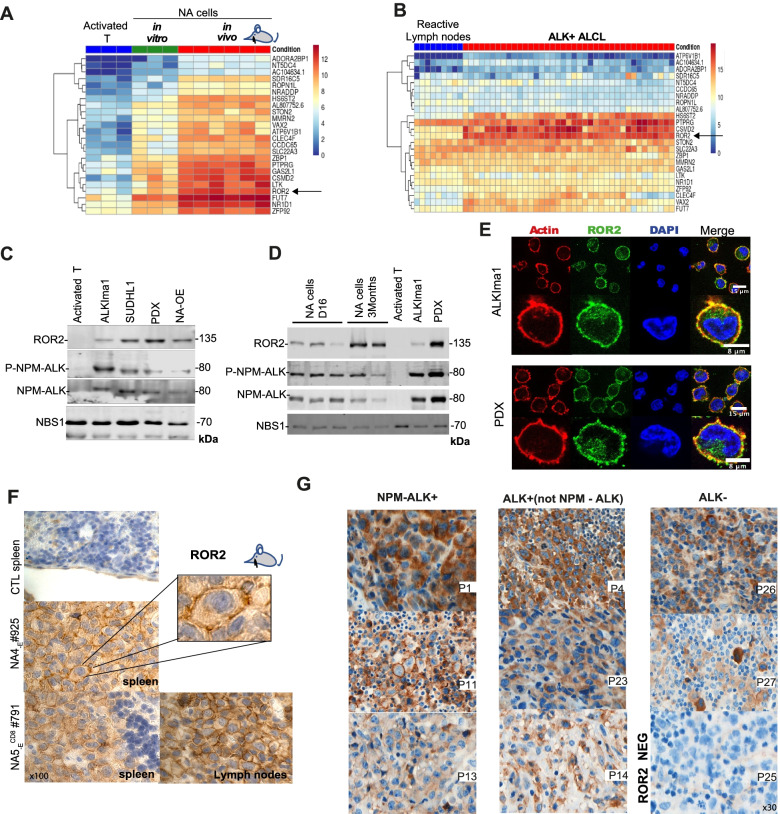
The ROR2 receptor is a robust ALK+ ALCL marker. **A.** Heatmap representation of genes showing progressive upregulation: from activated T lymphocytes to NA cells in vitro, then from NA cells in vitro to in vivo models (a threshold of twofold increased expression was applied for each assessment) in the model dataset. **B.** Heatmap representation of the list of genes found in **A** in the patient dataset (*n* = 39). Log2 transformed expression matrix was used to generate representations. **C.** Western blot analysis of ROR2, NPM-ALK and phosphorylated NPM-ALK (P-NPM-ALK) in activated T lymphocytes, ALKIma1 cells, an ALK+ ALCL cell line (SUDHL1), patient PDX cells and an NPM-ALK overexpression model (NA-OE). NBS1 was used as a loading control. **D.** Western blot analysis of ROR2, NPM-ALK and phosphorylated NPM-ALK (P-NPM-ALK) in NA cells 16 days and 3 months post-transfection in activated T lymphocytes, ALKIma1 cells, and patient PDX cells. NBS1 was used as a loading control. **E.** Immunofluorescence analysis of ROR2 expression in ALKIma1 and PDX patient cells. Green: ROR2, red: actin, blue: DAPI. **F.** Histologic analysis of a CD4+ spleen tumor (corresponding to mouse #925 injected with NA4_-E_) and a CD8+ spleen and lymph node tumors (corresponding to mouse #791 injected with NA5_-E_^CD8^): anti-ROR2 IHC. The negative control spleen (CTL) was obtained from a wild type NSG mouse. **G.** Histologic analysis of ALCL patient tumors: anti-ROR2 IHC. Left: NPM-ALK+ ALCL tumors (P1, P11, P13), middle: ALK+ ALCL tumors (other fusion partners) (P4, P23 and P14); right: ALK- ALCL tumors (P26 and P27); ROR2 negative tumor (P25). See also Table 1 for all sample data

We hypothesized that genes significantly upregulated during the early steps of transformation (in vitro NA models) and further upregulated in fully transformed cells (in vivo NA models) would provide insight into key pathological pathways and potential therapeutic targets. To identify such upregulated genes, we applied a twofold enrichment threshold to compare in vitro NA cells vs. activated T lymphocytes and a subsequent twofold threshold to compare in vivo vs. in vitro NA cells. Accordingly, we identified 24 genes that were consistently upregulated upon transformation (Fig. 6A**)**. Importantly, expression of these 24 genes efficiently distinguished ALK+ ALCL patients apart from controls (Fig. 6B), thus demonstrating that this molecular signature established from our models was relevant to identify ALK+ ALCL samples. The transmembrane ROR2 surface receptor was identified as clearly upregulated (Fig. 6A, B and Supplementary Fig. S7E). ROR2 protein is nearly absent from adult tissues [29] and only weakly expressed in activated T lymphocytes (Fig. 6A to D). Using western blot analysis, we confirmed ROR2 expression in ALK+ ALCL in the ALKIma1 cell line and various ALK+ ALCL cells, including a PDX model (Fig. 6C). Interestingly, NA cells expressed ROR2 at early stages during transformation (at 16 days, Fig. 6D). Immunofluorescence experiments show that ROR2 is markedly expressed at the membrane in the ALKIma1 cell line and PDX patient cells (Fig. 6E). As shown from our RNAseq data, all patient samples (39/39) expressed significantly increased levels of ROR2 (Fig. 5H, 6B). ROR2 is implicated in the WNT pathways and has been reported to drive both the canonical and non-canonical WNT pathways in cancer cells [30]. Interestingly, the WNT5B, WNT7B and WNT11 ligands were also upregulated in patients (Fig. 5E), which positively correlated with ROR2 expression levels (Supplementary Fig. S7F).

As ALCL diagnosis is largely based on histological analysis, we performed IHC assessment of ROR2 expression in spleen and lymph node sections derived from our NA models. We observed robust ROR2 expression with marked membrane expression (Fig. 6F, Supplementary Fig. S7G). We performed ROR2 IHC on biopsies from two independent cohorts of ALCL patient samples (cohort #1: P1 to P27 and cohort #2: P28 to P44) for which the ALK status was established using IHC and/or RNAseq. The samples were derived from an unselected cohort of ALCL patients (P1-P27) or from a cohort of patients with aggressive refractory disease (P28-P44). The analysis revealed positive expression of ROR2 in 40/44 patient samples (Table 1). In agreement with our expression data, nearly all ALK+ ALCL samples were ROR2 positive (37/38), with clearly discernible expression in the membrane. A large proportion of tumor cells expressed ROR2 (often 80 to 100% for 30/38 samples) and expression intensity was ranked from null (0) to high (3)(Fig. 6G, Table 1 and Supplementary Fig. S7H). All chemotherapy-resistant patient samples displayed ROR2 expression. Furthermore, ROR2 expression was observed in four patient samples harboring alternative ALK fusions with other or unknown ALK partners (Fig. 6G and Table 1), thus indicating that ROR2 is a genuine marker of ALK+ ALCL regardless of the fusion partner. Finally, three of the six tumor samples that did not express ALK were positive for ROR2, including two of four ALK- ALCL samples, thus indicating that aberrant ROR2 expression represents a marker for other types of ALK- human lymphoma (Fig. 6G and Table 1).

**Table 1:** IHC analysis of ROR2 in patient samples. Tumor samples: P1 to P27 (cohort #1), P28 to P44 and controls 1 to 3 (cohort #2). The table indicates diagnosis, ALK status, the ALK fusion partner gene (when identified), percentage of tumor cells positive for ROR2, ROR2 staining intensity [from 0 (null) to 3 (high)] and cellular sublocalization of ROR2 (cyto = cytoplasm; membr = membrane). CT: common type, SC: small cell; LH: lymphohistiocytic; HL: Hodgkin-like; T-NOS: T lymphoma not otherwise specified, NA: non-applicable, CTL: Control samples

Cohort #1						
**PATIENTS** (Fig. 6G)	**Diagnostic**	**ALK**	**ALK** **partner**	**ROR2 intensity**	**Percentage of tumoral cells expressing ROR2 (%)**	**Localization**
**P1**	**ALCL CT**	**pos**	**NPM**	**3**	**100**	**Cyto**
**P2**	**ALCL CT + SC**	**pos**	**non NPM**	**1**	**80**	**Membr**
**P3**	**ALCL CT**	**pos**	**NPM**	**3**	**80**	**Cyto**
**P4**	**ALCL CT + SARC**	**pos**	**non NPM**	**3**	**100**	**Cyto**
**P5**	**ALCL CT**	**pos**	**NPM**	**3**	**100**	**Membr**
**P6**	**ALCL LH**	**pos**	**NPM**	**1**	**40**	**Cyto**
**P7**	**ALCL SC + CT**	**pos**	**NPM**	**2**	**100**	**Membr**
**P8**	**ALCL CT**	**pos**	**NPM**	**2**	**70**	**Cyto**
**P9**	**ALCL CT**	**pos**	**NPM**	**2**	**100**	**Cyto**
**P10**	**ALCL CT**	**pos**	**NPM**	**2**	**100**	**Membr**
**P11**	**ALCL CT**	**pos**	**NPM**	**3**	**100**	**Membr + Cyto**
**P12**	**ALCL HD + SC**	**pos**	**NPM**	**2**	**100**	**Membr**
**P13**	**ALCL CT**	**pos**	**NPM**	**1**	**100**	**Membr**
**P14**	**ALCL CT**	**pos**	**ALO17**	**2**	**100**	**Membr**
**P15**	**ALCL CT**	**pos**	**NPM**	**2**	**90**	**Cyto**
**P16**	**ALCL CT**	**pos**	**NPM**	**1**	**100**	**Cyto**
**P17**	**ALCL CT**	**pos**	**NPM**	**1**	**NA**	
**P18**	**ALCL SC + CT**	**pos**	**NPM**	**2**	**90**	**Membr**
**P19**	**ALCL SC**	**pos**	**NPM**	**1**	**50**	**Membr**
**P20**	**ALCL CT**	**pos**	**NPM**	**2**	**NA**	**Cyto**
**P21**	**ALCL CT**	**pos**	**NPM**	**2**	**90**	**Membr + Cyto**
**P22**	**ALCL CT + SC + LH**	**pos**	**NPM**	**1**	**40**	**Cyto**
**P23**	**ALCL CT**	**pos**	**TFG**	**3**	**100**	**Cyto**
**P24**	**ALCL SC**	**pos**	**NPM**	**0**	**0**	**NA**
**P25**	**ALCL CT**	***neg***	**0**	**0**	**0**	**NA**
**P26**	**ALCL CT**	***neg***	**0**	**3**	**90**	**Cyto**
**P27**	**T-NOS**	***neg***	**0**	**2**	**100**	** Membr + Cyto**
Cohort #2						
**PATIENTS**	**Diagnostic**	**ALK**		**ROR2 intensity**	**Percentage of tumoral cells expressing ROR2 (%)**	**Localization**
**P28**	**ALCL**	**pos**		**3**	**100**	**Membr + Cyto**
**P29**	**ALCL**	**pos**		**2**	**80**	**Membr**
**P30**	**ALCL**	**pos**		**1**	**50**	**Membr**
**P31**	**ALCL**	**pos**		**1to 2**	**90**	**Membr**
**P32**	**ALCL**	**pos**		**2**	**100**	**Membr + Cyto**
**P33**	**ALCL**	**pos**		**3**	**70**	**Membr + Cyto**
**P34**	**ALCL**	**pos**		**3**	**80**	**Membr**
**P35**	**ALCL**	**pos**		**1 to 3**	**100**	**Cyto**
**P36**	**ALCL**	**pos**		**3**	**70**	**Membr + Cyto**
**P37**	**ALCL**	**pos**		**3**	**80**	**Membr + Cyto**
**P38**	**ALCL**	***pos***		**2**	**90**	**Membr + Cyto**
**P39**	**ALCL**	***pos***		**3**	**100**	**Membr + Cyto**
**P40**	**ALCL**	***pos***		**3**	**100**	**Membr + Cyto**
**P41**	**ALCL**	***pos***		**2**	**100**	**Membr + Cyto**
**P42**	**Large cell NHL CD30+**	***neg***		**0**	**0**	**0**
**P43**	**ALCL**	***neg***		**0**	**0**	**0**
**P44**	**ALCL**	***neg***		**2**	**50**	**Membr**
**Control samples**						
***CTL1***	***Non tumoral lymph nodes***	***neg***		***0***	***0***	***0***
***CTL2***	***Non tumoral lymph nodes***	***neg***		***0***	***0***	***0***
***CTL3***	***Nnon tumoral lymph nodes***	***neg***		***0***	***0***	***0***

According to the human expression data from the human cell atlas immune system (www.Immgen.org), we confirmed that a variety of immune cells express low levels of ROR2 (Supplementary Fig. S8A). Reanalysis of published ROR2 expression datasets (GSE6338, GSE14879, GSE19069 and GSE65823)) displayed higher ROR2 expression levels in ALK+ ALCL samples compared with other peripheral T-cell lymphoma samples (Supplementary Fig. S8B to D). We hypothesized that samples in the dataset GSE19069, which are characterized by reduced ROR2 expression levels in ALK+ ALCL, were comprised of tissues with fewer tumor cell infiltrates (Supplementary Fig. S8D). Further supporting our ALK- ALCL histology data, increased ROR2 expression was recurrently found in other independent ALK- ALCL patient samples (Supplementary Fig. S8 D to F) and in a few other T cell lymphomas (Supplementary Fig. S8D, E). In particular, an independent dataset displayed > 50% of ALK- ALCL with high ROR2 expression without concomitant higher ALK expression levels (Supplementary Fig. S8E), further indicating that ROR2 is an independent marker for human ALCL.

Overall, these results demonstrate that the molecular characterization of the evolution from normal T lymphocytes to in vivo NA models enabled the identification of ROR2 as a relevant cell surface marker of ALK+ ALCL tumor cells.

## Discussion

De novo generation of the pathognomonic t(2;5)(p23;q35) translocation presents significant advantages over other overexpression model approaches, as fusion oncogene expression is controlled by the human chromatin environment and subjected to endogenous regulatory elements upon transformation. These NA models provide a reliable framework to analyze cells from the initial process of oncogenic chromosomal rearrangement including the early steps of transformation, with pre-transformed cells as early as 15 days (Fig. 1B, C).

Generation of the NPM-ALK translocation enabled reproduction of the heterogeneous ALK+ ALCL tumor phenotype. Interestingly, the panel of independent ALK+ ALCL tumors (early injections in mice) did not always yield in vitro stable NA cell lines (Supplementary Table S1). While in vitro NA cells were maintained independently with various CD30 levels, all tumors expressed high levels of CD30. These data indicate a strong positive in vivo selection of CD30. Interestingly, expression data from CD3+ and CD3- populations within the same tumor revealed that cell cycle genes were enriched in CD3- cells, which is associated with increased proliferative activity rather than a differentiation state. Furthermore, we observed CD8+ tumors that reflected the oncogenic potential of the NPM-ALK-associated translocation in CD8+ T lymphocytes.

Additionally, these models displayed several other clinical features observed in ALK+ ALCL patients. Indeed, patients most often develop tumors in lymph nodes, followed by extranodal sites (60%) and typical skin lesions (up to 25% for pediatric ALK+ ALCL) [1, 2]. Accordingly, mice injected with NPM-ALK translocated cells developed tumors in lymph nodes, splenomegaly, lung infiltrations and skin nodules. Finally, tumor cells from NA models also recapitulated the various cellular morphologies observed in patients including large ALCL common type cells, with a group of small cells. Of note, consistent with the lack of recurrent additional genetic alteration reported in ALK+ ALCL patients, analysis of variants using RNAseq data from our NA murine tumors did not identify obvious additional driver events using the Cancer Genome Interpreter. Indeed, while some putative driver events were found, it is worth noting that none of the alterations are recurrent and most of the variants correspond to known SNP listed in dbSNP or other databases (see Supplementary Table S10). These results suggest that recurrent additional mutation is not required for NPM-ALK tumorigenesis in these models.

Molecular analysis of these models revealed that the activation of both the canonical and non-canonical WNT pathways is relevant to ALK+ ALCL patient tumors. The WNT pathway, which coordinates multiple biological processes such as development, stemness, tissue regeneration and homeostasis, has been associated with metastasis, cancer stem cells and immune control [31–34]. Simultaneous activation of the two WNT pathways has been reported in colon cancer cells [35], and the WNT/β-catenin pathway is upregulated in a highly tumorigenic subpopulation of ALK+ ALCL cell lines [36]. Our data further indicate that an imbalance in WNT pathway activation occurs in ALK+ ALCL. Evidence has shown that the WNT pathways acts in oncogenic signaling to promote immune evasion in tumors [37, 38]. Overactivation of the WNT pathway in ALK+ ALCL tumors could inhibit the activity of therapeutics, such as anti-PD1 therapy (now in clinical trials) as suggested for melanoma treatment [37]. Furthermore, cancer-specific WNT inhibitors are under development [39] and could benefit ALK+ ALCL patients.

Analyzing our models, we identified a gene signature of 24 upregulated genes that efficiently distinguished ALK+ ALCL patient samples from controls. The ROR2 membrane receptor was highly upregulated in 100% of patient samples, which was validated by histological assessment. ROR2 has been shown to act in both canonical and non-canonical pathways [30]. Notably, we found that several WNT ligands were upregulated in ALK+ ALCL cells, which positively correlated to ROR2 expression levels (Fig. 5E, Supplementary Fig. S7F). WNT5b and WNT11 have been shown to regulate the non-canonical WNT pathway in non-hematological cancers (for review see [32]). WNT5B has been shown to interact with ROR2 in osteosarcoma [40], while preliminary results indicate that WNT11 could be a ligand of ROR2 in breast cancer [41]. These data suggest that alteration of both WNT ligands and their respective receptors may provide an autocrine mechanism to maintain lymphoma cells. ROR2 has been shown to control cell migration in mice and *C. elegans* [42, 43]. Overexpression of ROR2 in osteosarcoma [40], melanoma [44] and breast cancer [45] has been proposed to play a role in cell migration, cell proliferation and spatiotemporal control of tumor cell heterogeneity [46]. Indeed, deciphering the function of ROR2 and its relationship with WNT pathway activation in migration and metastatic processes would provide further insight into ALK+ ALCL pathophysiology.

ROR2 is a membrane receptor with a very restricted pattern of expression, which is increased during early embryogenesis and decreased during ontogeny to almost undetectable levels in most adult organs [30]. Thus, the distinct ROR2 expression pattern in ALK+ ALCL patient samples indicates that ROR2 and downstream oncogenic pathways may represent potential therapeutic targets. This study also highlights the potential to use ROR2 as an immune marker to target ALCL cells. In this regard, the current phase 1–2 trials of ADC antibody and CART targeting ROR2 for other tumor types (e.g., NSCLC and ovarian tumors: ClinicalTrials.gov #NCT03504488, NCT03960060, NCT03393936, [30]) may also benefit ALK+ ALCL patients.

## Conclusion

In conclusion, this work demonstrates that ab initio modeling of NPM-ALK specific chromosomal translocation in mature T lymphocytes provides unique models to identify novel therapeutic targeting approaches. Our model enabled the identification of new candidate genes. Remarkably, we found that ROR2 may represent a genuine biomarker of ALK+ ALCL that may be targeted to treat patients.

## Supplementary Information

**Additional file 1.** Supplementary Data.

## Data Availability

RNA-seq data generated in this study were deposited in the ArrayExpress database (E-MTAB-10924). RNA-seq data for ALCL patients were previously published [7]. Other raw data from this study are available on Mendeley reserved DOI: doi:10.17632/x5fyb55w9x.1

## Abbreviations

ALK: Anaplastic lymphoma kinase
ALK+ ALCL: Anaplastic large cell lymphoma ALK positive
NA cells: Cells positive for the NPM-ALK-associated translocation
PBMC: Peripheral blood mononuclear cell
CRISPR: Clustered Regularly Interspaced Short Palindromic Repeats
Cas9: CRISPR-associated protein 9
gRNA: Guide RNA
