# Supplementary material for: De novo generation of the NPM-ALK fusion recapitulates the pleiotropic phenotypes of ALK+ ALCL pathogenesis and reveals the ROR2 receptor as target for tumor cells
Source: Mol Cancer. 2022 Mar 4;21:65. doi: 10.1186/s12943-022-01520-0 (PMC8895835; doi:10.1186/s12943-022-01520-0)
Supplement: Supplementary file 1 — Additional file 1. Supplementary Data. [file 12943_2022_1520_MOESM1_ESM.zip › Additional file/SupplementaryData-MolCancer-Revised.docx]

**SUPPLEMENTARY DATA**

**Supplementary Methods**

**CD4+ cell sorting**

PBMCs were washed twice with PBE (PBS, 0,5% BSA, 2mM EDTA) and ells were incubated at 4°C for 20 minutes with 5 µL anti CD4-PE (Miltenyi Biotec #130-113-254 RRID: AB_2726056). Cells were washed twice with PBE and incubated at 4°C for 20 minutes with 10 µL Anti-PE MicroBeads (Miltenyi Biotec #130-048-801, RRID: AB_244373) then purified on LS columns (Miltenyi Biotec #130-042-401) following manufacturer’s instructions.

**Flow cytometry and cell viability**

Cells were stained with antibodies using standard protocols and analyzed by flow cytometry (Fortessa system, BD Biosciences). Antibodies are described in below. Post-acquisition analyses were performed using FlowJo software. Cell viability was assessed using LIVE/DEAD™ Fixable Far Red Dead Cell Stain Kit (Invitrogen #L34973) following manufacturer’s instructions.

**FACS conjugated antibodies (in vitro experiments):**

CD8-FITC: anti-human CD8a Clone RPA-T8 Sony ; CD8-BV650 : #344730 Biolegend; CD4-APC : #357408 Biolegend; CD4-BV510 : #100559 Biolegend; CD30-PE : #550041 BD Bioscience; CD4-BV421 : #2387120 Sony; CD3-BV785 : #2186650 Sony

**FACS conjugated antibodies (in vivo experiments):**

CD3-BV510 (clone UCHT1) : #563109 BD Bioscience; CD3-FITC (Clone UCHT1) : #11-0038-42 invitrogen; CD3-PE (Clone UCHT1) : #5204501 BD Bioscience; CD4-FITC (Clone RPA-T4) : #11-0049-42 eBiosciences; CD8-PE (Clone RPA-T8) : #555367 BD Bioscience; CD30-PE (Clone BerH8) : #550041 BD Bioscience; CD45-APC-Cy7 (Clone HI30) : #47-0459-42 invitrogen; CD274(PDL-1)-PE-Cy7 (Clone MIH1) : #25-5983-42 eBiosciences; CD3 : #M725401 Dako ; CD4 : #ab213215 abcam ; CD8 : #M3164 Spring

**G1/S checkpoint analysis**

Cells were γ−irradiated at 5Gy. After 14h, 10 µM of BrdU (Merck #B5002) was added in the media. Cells were resuspended in PBS-BSA 1% and fixed with cold ethanol 70%. After overnight incubation, cells were centrifuged at 4°C, 10 min at 1500 rpm then resuspended in 2N HCl/0.5% Triton X100 for 30 min at RT. Similarly, cells were centrifuged and resuspended in tetraborate 0,1M pH8.5 for 10 min at RT. Finally, pellets were resuspended in 50 µL PBS-BSA 1% 0.5% Tween with 10 µL of anti-BrdU FITC (BD Pharmingen #347583 RRID: AB_400327) for 30 min at RT. Flow cytometry analyses were done with Propidium Iodide 50 mg/mL (Sigma #P4864) (Gallios (Beckman Coulter)). Results were analyzed with FlowJo software.

**Western blotting**

Whole-cell extracts were prepared with protein lysis buffer (50 mM Tris-HCl pH 7.4, 1%Triton X-100, 0.1% SDS, 150 mM NaCl, 1 mM EDTA, and 1 mM DTT), supplemented with « Complete cocktail protease inhibitor tablets » (Roche #11697498001) and « Halt Protease and Phosphatase Inhibitor Cocktail » (Thermo Fisher Scientific #78440). Typically, 30 μg of protein were loaded on 8% acrylamide gel (w/v) Tris-HCl SDS PAGE. After transfer, membranes were stained with anti-Phospho-ALK (Tyr1604 #3341 RRID: AB_331047), anti-ALK (31F12 #3791, RRID: AB_1950402), anti-P-STAT3 (Tyr705 #9131, RRID: AB_331586), anti-STAT3 (#4904, RRID: AB_331269) anti-NPM1 (#3542, RRID: AB_2155178) from Cell Signaling, and anti-P53 (#sc-126, RRID: AB_628082); anti-ROR2 (#sc-374174, RRID: AB_10989358) from Santa Cruz Biotechnology. Anti-Vinculin (#sc-73614 Santa Cruz Biotechnology, RRID: AB_1131294) and anti-NBS1 (#NB100-143 Novus, RRID: AB_10078050) antibodies were used as loading controls. Secondary antibodies IRDye 800CW and IRDye  680RD were used, and revelation was performed using « Odyssey® CLx Imaging System » (LI-COR).

**Fluorescence in situ hybridization and conventional cytogenetic analysis**

FISH was performed on metaphases from translocated T cells. Metaphase spreading, probe labeling, hybridization, washing and fluorescence detection were performed according to standard procedures. FISH was performed on the metaphases using ALK Break-apart #LPS 019 probe (Cytocell) and homemade NPM1 probe from BAC RP11-546B8 to detect the NPM1-ALK chromosomal translocation. Cell images were captured using a ZEISS AxioImager.Z2 microscope. For conventional karyotypes, metaphase spreads, R‐banded chromosomes were analyzed by standard procedures.

**Immunofluorescence analysis ROR2 and beta catenin immunofluorescence analysis**

Typically, 500 000 cells (ALKIma1) were seeded on glass cover slip previously coated with poly-L-lysine for 5 minutes at room temperature. After 1 hour of incubation at 37°C to allow cell adhesion, samples were fixed for 10 minutes at RT with 4% paraformaldehyde in PBS. After fixation, cells were permeabilized with 0,5% Triton X-100 for 10 minutes and blocked with 500uL 1% BSA in PBS for 30 minutes. Cells were stained with anti-ROR2 antibody (1:500, Santa Cruz #sc374174) or anti-beta-catenin antibody (1:200, R&D Systems #AF1329) diluted in 1% BSA/1X PBS overnight at 4°C. After 2 washes with 1% BSA/1X PBS, cells were incubated with Alexa Fluor 488-conjugated donkey anti-mouse (1:1000, Invitrogen, #A21202) and tetramethylrhodamine B isothiocyanate-conjugated phalloidin (1:100, Sigma, #P1951) diluted in 1% BSA/1X PBS for 1 hour. Coverslips were then counterstained with DAPI mounted in Vectashield Antifade Mounting Media (Vector Laboratories). Samples were imaged using a ZEISS AxioImager.Z2 microscope.

**Telomerase Repeat Amplification Protocol (TRAP)**

Fresh cells were resuspended in 100 µL of CHAPS Lysis Buffer and TRAP assay was performed following the manufacturer’s instructions of TRAPeze Telomerase Detection Kit (#S7700, Millipore). PCR products were run on a TBE/acrylamide:bisacrylamide (19:1) gel, stained with SYBR Gold Nucleic Acid Gel Stain (Invitrogen) and visualized with a FLA-3000 Phosphorimager (Fujifilm).

**Microarray analyses of** datasets (GSE6338, GSE14879, GSE19069 and GSE65823)

Three datasets (GSE6338, GSE14879, GSE19069 and GSE65823) were analyzed in order to investigate the level of expression of ROR2. All datasets were similarly analyzed. To obtain detailed information about experimental conditions associated with samples, we used getGEO (GEOquery R package v2.58.0) using the path of the .soft.gz file (value of the argument = filename). Conditions associated to each sample of the dataset of interest were retrieved using the following R command line (require the magrittr package): lapply(training.geo@gsms,function(z){z@header$title}) %>% unlist(). Data contained in .CEL files were retrieved and stored in a R object using the function ReadAffy (affy package v1.68.0). Normalization between samples was then performed with the function rma (affy package) with default values for all parameters. The design matrix was generated using the function model.matrix (package stats v4.0.3). The value of the argument object of the function was the following model formula : ~ 0 + groups (groups correspond to the vector of the class vector that gives the condition associated with each sample). A linear model was then fit for each probe using the function lmFit (package limma 3.46.0) with the ExpressionSet object containing the normalized matrix as value of the argument object and the design matrix generated before as value of the argument design. To identify differential probes between conditions, a contrast matrix was firstly built using the function makeContrasts (limma). The desired comparisons (between two conditions) were specified as input of the function. The design matrix was given as the value of the argument levels of the function. The estimation of coefficients and standard errors for the set of contrasts specified before was performed with the function contrasts.fit (limma) with the linear model fitting to the microarray data previously generated as value of the argument fit and the contrast matrix as value of the argument contrast.matrix. Moderated t-statistics, F-statistic and log-odds of differential expression were computed by empirical Bayes moderation of the standard errors towards a global value using the function eBayes (limma) using the result of the function contrasts.fit as value of the argument fit of this function. To identify genes that can be considered as differentially expressed after adjusting for multiple testing the function decideTests (limma) was used with the result of the function eBayes as value of the argument object. Values given of other arguments of the function are : method='global',adjust.method="BH",p.value=0.05,lfc=1. Plots were performed using ggplot2 (v3.3.3). The probe associated with ROR2 in each dataset investigated is : 205578_at (Affymetrix Human Genome U133 Plus 2.0 Array).

**Primers sequences**

**Translocation detection**

Der2-F : GAGACATGCCCAGGACAGAT

Der2-B : AGAGCACATGGGAAAAGGAA

Der5-NF : GAACTCCTGGCCTTAACGTG

Der5-NB : CCACCCTCTAGGGTTGTCAAT

**hTERT promoter**

hTERT-Prom-F : CACCTTCCAGCTCCGCCT

hTERT-Prom-B : CAGCTCCTTCAGGCAGGACAC

**Breakpoint junctions sequencing by NGS:**

Der2-NGS-F : GCAGCGTCAGATGTGTATAAGAGACAG TACGTGCTCGGCAATTTACA

Der2-NGS-B : TGGGCTCGGAGATGTGTATAAGAGACAG TGGAGTGTGCCTGTAGTTCTAGC

Der5-NGS-F : GCAGCGTCAGATGTGTATAAGAGACAG GCTGGAGTGCAGTGATGTGA

Der5-NGS-B : TGGGCTCGGAGATGTGTATAAGAGACAG TTCCCTCCTCTATGCAATGG

**NGS adapter primers:**

BC1 CAAGCAGAAGACGGCATACGAGATACGGATTCGTCTCGTGGGCTCGGAGATGT

AATGATACGGCGACCACCGAGATCTACACCAACTCCATCGTCGGCAGCGTCAGATGT

BC2 CAAGCAGAAGACGGCATACGAGATTGCTCATGGTCTCGTGGGCTCGGAGATGT

AATGATACGGCGACCACCGAGATCTACACGATCTTGCTCGTCGGCAGCGTCAGATGTG

BC3 CAAGCAGAAGACGGCATACGAGATGTCCTAAGGTCTCGTGGGCTCGGAGATG

AATGATACGGCGACCACCGAGATCTACACCTTCACTGTCGTCGGCAGCGTCAGATGTG

BC4 CAAGCAGAAGACGGCATACGAGATGGTCAGATGTCTCGTGGGCTCGGAGATGT

AATGATACGGCGACCACCGAGATCTACACCTCGACTTTCGTCGGCAGCGTCAGATGTG

BC5 CAAGCAGAAGACGGCATACGAGATTCGGTTACGTCTCGTGGGCTCGGAGATGT

AATGATACGGCGACCACCGAGATCTACACAAGACACCTCGTCGGCAGCGTCAGATGTG

BC6 CAAGCAGAAGACGGCATACGAGATGTACCTTGGTCTCGTGGGCTCGGAGATGT

AATGATACGGCGACCACCGAGATCTACACCGTATCTCTCGTCGGCAGCGTCAGATGTG

BC7 CAAGCAGAAGACGGCATACGAGATAGACCTTGGTCTCGTGGGCTCGGAGATGT

AATGATACGGCGACCACCGAGATCTACACTTACGTGCTCGTCGGCAGCGTCAGATGT

BC8 CAAGCAGAAGACGGCATACGAGATGTAACGACGTCTCGTGGGCTCGGAGATGT

AATGATACGGCGACCACCGAGATCTACACAGCTAAGCTCGTCGGCAGCGTCAGATGTG

BC9 CAAGCAGAAGACGGCATACGAGATGAGATACGGTCTCGTGGGCTCGGAGATGT

AATGATACGGCGACCACCGAGATCTACACCAAGGTACTCGTCGGCAGCGTCAGATGTG

BC10 CAAGCAGAAGACGGCATACGAGATGCACGTAAGTCTCGTGGGCTCGGAGATGT

AATGATACGGCGACCACCGAGATCTACACAGACCTTGTCGTCGGCAGCGTCAGATGTG

BC11 CAAGCAGAAGACGGCATACGAGATGCTTAGCTGTCTCGTGGGCTCGGAGATGT

AATGATACGGCGACCACCGAGATCTACACGTCGTTACTCGTCGGCAGCGTCAGATGTG

BC12 CAAGCAGAAGACGGCATACGAGATGGTGTCTTGTCTCGTGGGCTCGGAGATGT

AATGATACGGCGACCACCGAGATCTACACGTAACCGATCGTCGGCAGCGTCAGATGTG

BC13 CAAGCAGAAGACGGCATACGAGATTGGAGTTGGTCTCGTGGGCTCGGAGATGT

AATGATACGGCGACCACCGAGATCTACACGAATCCGTTCGTCGGCAGCGTCAGATGTG

BC14 CAAGCAGAAGACGGCATACGAGATGCAAGATCGTCTCGTGGGCTCGGAGATGT

AATGATACGGCGACCACCGAGATCTACACCATGAGCATCGTCGGCAGCGTCAGATGTG

BC15 CAAGCAGAAGACGGCATACGAGATCAGTGAAGGTCTCGTGGGCTCGGAGATGT

AATGATACGGCGACCACCGAGATCTACACCTTAGGACTCGTCGGCAGCGTCAGATGTG

BC16 CAAGCAGAAGACGGCATACGAGATAAGTCGAGGTCTCGTGGGCTCGGAGATGT

AATGATACGGCGACCACCGAGATCTACACATCTGACCTCGTCGGCAGCGTCAGATGT

BC17 CAAGCAGAAGACGGCATACGAGATCGTATTCGGTCTCGTGGGCTCGGAGATGT

AATGATACGGCGACCACCGAGATCTACACTTACCGACTCGTCGGCAGCGTCAGATGTG

BC18 CAAGCAGAAGACGGCATACGAGATTCAAGGACGTCTCGTGGGCTCGGAGATGT

AATGATACGGCGACCACCGAGATCTACACTCGTCTGATCGTCGGCAGCGTCAGATGTG

BC19 CAAGCAGAAGACGGCATACGAGATAAGCACTGGTCTCGTGGGCTCGGAGATGT

AATGATACGGCGACCACCGAGATCTACACTTCCAGGTTCGTCGGCAGCGTCAGATGTG

BC20 CAAGCAGAAGACGGCATACGAGATGCAATGGAGTCTCGTGGGCTCGGAGATGT

AATGATACGGCGACCACCGAGATCTACACTACGGTCTTCGTCGGCAGCGTCAGATGT

BC21 CAAGCAGAAGACGGCATACGAGATCAATCGACGTCTCGTGGGCTCGGAGATGT

AATGATACGGCGACCACCGAGATCTACACAAGACCGTTCGTCGGCAGCGTCAGATGTG

BC22 CAAGCAGAAGACGGCATACGAGATGGCGTTATGTCTCGTGGGCTCGGAGATGT

AATGATACGGCGACCACCGAGATCTACACCAGGTTCATCGTCGGCAGCGTCAGATGTG

BC23 CAAGCAGAAGACGGCATACGAGATGTTAAGGCGTCTCGTGGGCTCGGAGATGT

AATGATACGGCGACCACCGAGATCTACACTAGGAGCTTCGTCGGCAGCGTCAGATGTG

BC24 CAAGCAGAAGACGGCATACGAGATCCTATACCGTCTCGTGGGCTCGGAGATGT

AATGATACGGCGACCACCGAGATCTACACTACTCCAGTCGTCGGCAGCGTCAGATGTG

**gRNA sequences**

gRNA^NPM1^ : TATATCCTCGAACTGCTACT

gRNA^ALK^  : GATCAGATTAGGGTTACCTG

*gRNAs additional sequences used in SupFig S1B:*

gRNA^NPM1^ : GTGAACCCAGTAGCAGTTCG

gRNA^ALK^ : GTCGGTCCATTGCATAGAGG

**Supplementary References:**

1. Piccaluga PP, Agostinelli C, Califano A, Rossi M, Basso K, Zupo S, Went P, Klein U, Zinzani PL, Baccarani M, et al: **Gene expression analysis of peripheral T cell lymphoma, unspecified, reveals distinct profiles and new potential therapeutic targets.** *J Clin Invest* 2007, **117:**823-834.

2. Crescenzo R, Abate F, Lasorsa E, Tabbo F, Gaudiano M, Chiesa N, Di Giacomo F, Spaccarotella E, Barbarossa L, Ercole E, et al: **Convergent mutations and kinase fusions lead to oncogenic STAT3 activation in anaplastic large cell lymphoma.** *Cancer Cell* 2015, **27:**516-532.

3. Eckerle S, Brune V, Doring C, Tiacci E, Bohle V, Sundstrom C, Kodet R, Paulli M, Falini B, Klapper W, et al: **Gene expression profiling of isolated tumour cells from anaplastic large cell lymphomas: insights into its cellular origin, pathogenesis and relation to Hodgkin lymphoma.** *Leukemia* 2009, **23:**2129-2138.

4. Giefing M, Winoto-Morbach S, Sosna J, Doring C, Klapper W, Kuppers R, Bottcher S, Adam D, Siebert R, Schutze S: **Hodgkin-Reed-Sternberg cells in classical Hodgkin lymphoma show alterations of genes encoding the NADPH oxidase complex and impaired reactive oxygen species synthesis capacity.** *PLoS One* 2013, **8:**e84928.

5. Weniger MA, Tiacci E, Schneider S, Arnolds J, Ruschenbaum S, Duppach J, Seifert M, Doring C, Hansmann ML, Kuppers R: **Human CD30+ B cells represent a unique subset related to Hodgkin lymphoma cells.** *J Clin Invest* 2018, **128:**2996-3007.

6. Iqbal J, Weisenburger DD, Greiner TC, Vose JM, McKeithan T, Kucuk C, Geng H, Deffenbacher K, Smith L, Dybkaer K, et al: **Molecular signatures to improve diagnosis in peripheral T-cell lymphoma and prognostication in angioimmunoblastic T-cell lymphoma.** *Blood* 2010, **115:**1026-1036.

7. Agnelli L, Mereu E, Pellegrino E, Limongi T, Kwee I, Bergaggio E, Ponzoni M, Zamò A, Iqbal J, Piccaluga PP, et al: **Identification of a 3-gene model as a powerful diagnostic tool for the recognition of ALK-negative anaplastic large-cell lymphoma.** *Blood* 2012, **120:**1274-1281.

8. Pomari E, Basso G, Bresolin S, Pillon M, Carraro E, d'Amore ES, Viola G, Frasson C, Basso K, Bonvini P, Mussolin L: **NPM-ALK expression levels identify two distinct subtypes of paediatric anaplastic large cell lymphoma.** *Leukemia* 2017, **31:**498-501.

**SUPPLEMENTARY Files**

**Supplementary Tables**

**Table S1:**

Description of the independent experiments (donor 1 to 15) and corresponding mice experiments.

**Table S2:**

Results of NGS sequencing (number of reads) for each type of breakpoint junctions (Day 5 and 15) for donors 1 to 4 describing (indels sizes and sequence, microhomologies motifs (MH)).

**Tables S3 and S4**

Analysis of differentially expressed genes between CD3- and CD3+ cells (in vivo NA models) and GSEA analysis

**Tables S5 to S9**

Data of the GSEA analysis for in vitro NA models ,in vivo NA models and patient cells with the commonly enriched gene sets (S8) and extracellular matrix genes (S9).

**Tables S10**

Analysis of variants using RNAseq data from our NA mouse tumors

**Supplementary Figure Legends**

**Figure S1: Generation of NA cells derived from activated T lymphocytes for other donors**

1. Survival curve of control T lymphocytes (CTL: CD4+, CD8+ or unsorted cells, lines in orange) and NA cells (CD4+, CD8+ or unsorted cells, in purple) after CRISPR/Cas9 transfection.
2. Frequency of translocation detected by PCR amplification of Der5 and Der2 at 15 days post-transfection using a second pair of gRNAs targeting *NPM1* and *ALK* (Donor 1, 2 and 4). PCR were performed in duplicate on DNA dilutions (dilutions : 25 ng to 0.1 ng). Translocation frequency (F) is calculated using the assumption that a human diploid cell contains ~6 pg of DNA.
3. Example of Der2 and Der5 sequences obtained by Sanger sequencing from NA cells at 2.5 months post transfection for Donors 2 to 4.
4. Telomerase activity assessed by TRAP experiment of ALKIma1 cells. Activated T lymphocytes and telomerase negative cells (CLT-) were used as controls
5. Western blot against P53 in ALKIma1 cells. A T cell line, knock-out for P53, was used as negative control (CTL P53-/-). Vinculin was used as loading control.
6. G1/S checkpoint analysis by BrDU incorporation and FACS analysis in irradiated/non irradiated ALKIma1 cells. A T cell line, knock-out for P53 was used as control for p53 mediated G1/S checkpoint inactivation.
7. Tumor formation in mice after subcutaneous injection of ALKIma1 cells. On the right, skin nodules.
8. Histologic analysis of ALKIma1 tumor cells: anti-CD2, anti-CD5, anti-CD7, anti-CD20, anti-Granzyme B, and anti-Perforin on tumor from sous-cutaneous injections.

**Figure S2: Immuno-phenotypes of NA cells from activated T lymphocytes overtime**

1. Flow cytometry monitoring of CD4, CD8, CD3 and CD30 cells surviving overtime between 0 and 20 days post-activation, for primary activated T lymphocytes.
2. Flow cytometry monitoring of CD4, CD8, CD3 and CD30 cells surviving overtime between 0 and 15 days post-transfection, for NA cells transfected with gRNA*^NPM1^* and gRNA*^ALK^* .

**Figure S3: : Histological and immune phenotypes of tumors obtained from NA1 and NA2 cells**

1. Translocation detection by PCR amplification of derivative chromosomes Der2 and Der5 for NA1, NA2 and NA3 cells, 1 month post transfection.
2. Flow cytometry monitoring of CD45 and CD3 expression of cells from the blood of mice injected with NA1 and NA2 cells (46 days post injection, 4 different mice).
3. Analysis of mice after IV injection of NA cells. Left: Image of skin abnormalities for a mouse injected with NA1 cells compared to a control mouse (CTL). Right: spleen enlargement from mice injected with NA1 and NA2 cells compared to a normal spleen (CTL).
4. Histologic analysis of tumors in liver, spleen and lung from mice injected with NA1 cells: anti CD3 IHC compared to control organs (CTL).
5. Flow cytometry monitoring of CD45, CD30, PDL1, CD4 and CD3 expression in tumor cells (spleen and lymph node) obtained from NA1 and NA2 cells for different recipient mice (#150,#159, #175).
6. Flow cytometry monitoring of CD45, CD30, CD4, PDL1, and CD3 expression in tumors formed in secondary (NA1 IIr) recipient mice lymph node (mouse #418) and in spleen (mouse #443) obtained from NA1 cells.

**Figure S4 : Histological phenotype of skin tumors and TCR analysis of tumors obtained from NA2 cells**

1. Histologic analysis of tumor from NA2 cells in skin nodules: H&E staining, anti-CD30, anti-ALK, anti-CD4, anti-CD3 and anti-CD8.
2. TCR clonality analysis of NA1 and NA2 cells 1 month post-transfection.

**Figure S5: Immune phenotypes of tumors obtained from early injections**

1. Flow cytometry monitoring of CD4, CD8, CD3 and CD30 expression of NA-E cells.
2. Frequency of translocation detected by PCR amplification of derivative chromosome 5, Der5, 15 days after transfection for NA-E cells. PCR were performed in duplicate on DNA dilutions from 25 ng to 0.1 ng. Translocation frequency (F) is calculated using the assumption that a human diploid cell contains ~6 pg of DNA.
3. Flow cytometry monitoring of CD45 and CD3, CD4, CD8 expression of cells from the blood of different recipient mice injected with NA_-E_ cells (1.5 or 2 months post injection).
4. Flow cytometry monitoring of CD45, CD30, PDL1,CD4,CD3 and CD8 and of cells from tumors of different recipient mice injected with NA_-E_ cells (spleen and lymph node).

**Figure S6 : Histological phenotypes of tumors obtained from early injections of NA cells**

1. Histologic analysis of tumors in spleen, liver and lung from mice (NA4-E #923 and NA5-ECD8 #925): H&E staining, anti-CD4 and anti-CD8 IHC.
2. Derivative chromosome 5 (Der5) breakpoint sequences analyzed by Sanger sequencing from tumors obtained after injections with NA_-E_ cells.

**Figure S7 : Expression analysis of *in vivo* tumors obtained from NA cells**

1. Principal component analysis of the RNAseq data (Dimension 1 and 2) from CD3+ and CD3- tumor cells obtained from NA cells. Data were scaled to unit variance before performing the representation **(Supplementary Tables S3)**.
2. GSEA analysis showing enrichment in cell cycle gene sets in CD3- *in vivo* tumor cells compared to CD3+ *in vivo* tumor cells.
3. 29 commonly upregulated genes between in vitro NA models, in vivo NA models and patients in the gene list “Extracellular_matrix”
4. GSEA analysis of a gene list for the canonical and non-canonical WNT signaling pathways : NA cell *in vitro* and *in vivo* models vs. activated T lymphocytes expression data.
5. ROR2 gene expression (ddPCR) in activated T lymphocytes (WT) (in triplicates), NA cells *in vitro* (in triplicates) and NA cells *in vivo* (two triplicates).
6. Normalized expression levels (RNAseq) of WNT5B, WNT7B and WNT11 relative to ROR2 expression level found in control reactive lymph nodes and patient ALK+ ALCL with positive linear correlations reported for each WNT ligands.
7. Histologic analysis of lymph node tumors obtained from NA cells (NA-_E_^CD8^ #791): anti-ROR2 IHC.
8. Histologic analysis of ALCL patient tumors from the 2 patient cohortes: anti-ROR2 IHC. The intensity of expression level (1 to 3) and the percentage of tumor cells positive for ROR2 (% of ROR2+) are indicated. See also Table1 for other sample data

**Figure S8 : ROR2 expression in different ALK+ ALCL models**

1. ROR2 displays low expression across a variety of blood cells (data generated by Immgen.org).
2. ROR2 expression analysis (normalized expression level) in T cell lymphomas including ALCL compared to different types of T lymphocytes. The data are extracted from GSE6338 of Supplementary References [1, 2]. Statistical values are shown (t test).
3. ROR2 expression analysis (normalized expression level) in ALCL tumors (ALK positive or negative tumors but also for cutaneous ALCL), in different types of T and NK cells but also in ALCL cell lines (Karpas 299, SR786 and SUDHL1). In red: ALK- ALCL tumors with also high ROR2 expression. The data are extracted from GSE14879 of Supplementary Reference ([2-5]). Statistical values are shown (t test).
4. ROR2 expression analysis (normalized expression level) in different T lymphomas including ALCL (ALK positive or negative), in different types of T cells but also in acute lymphoblastic lymphoma cell lines (PEE, MOLT13, JURKAT). In red: tumor samples other than ALK+ ALCL with also high ROR2 expression. The data are extracted from GSE19069 of Supplementary Reference ([2, 6]). Statistical values are shown (t test).
5. ROR2 expression analysis (normalized expression level) in ALCL from [GSE65823](https://www.ncbi.nlm.nih.gov/geo/query/acc.cgi?acc=GSE65823) data from Supplementary Reference [7]. In red: ALK- ALCL tumors with also high ROR2 expression. Statistical values are shown (FDR).
6. ROR2 expression analysis (2logFold) for ALCL compared to normal tissues. Subgroup are labeled depending on their ALK expression level (data were analyzed using R2.amc.nk from the dataset GSE78513, Supplementary Reference [8])

**SUPPLEMENTARY FIGURES**


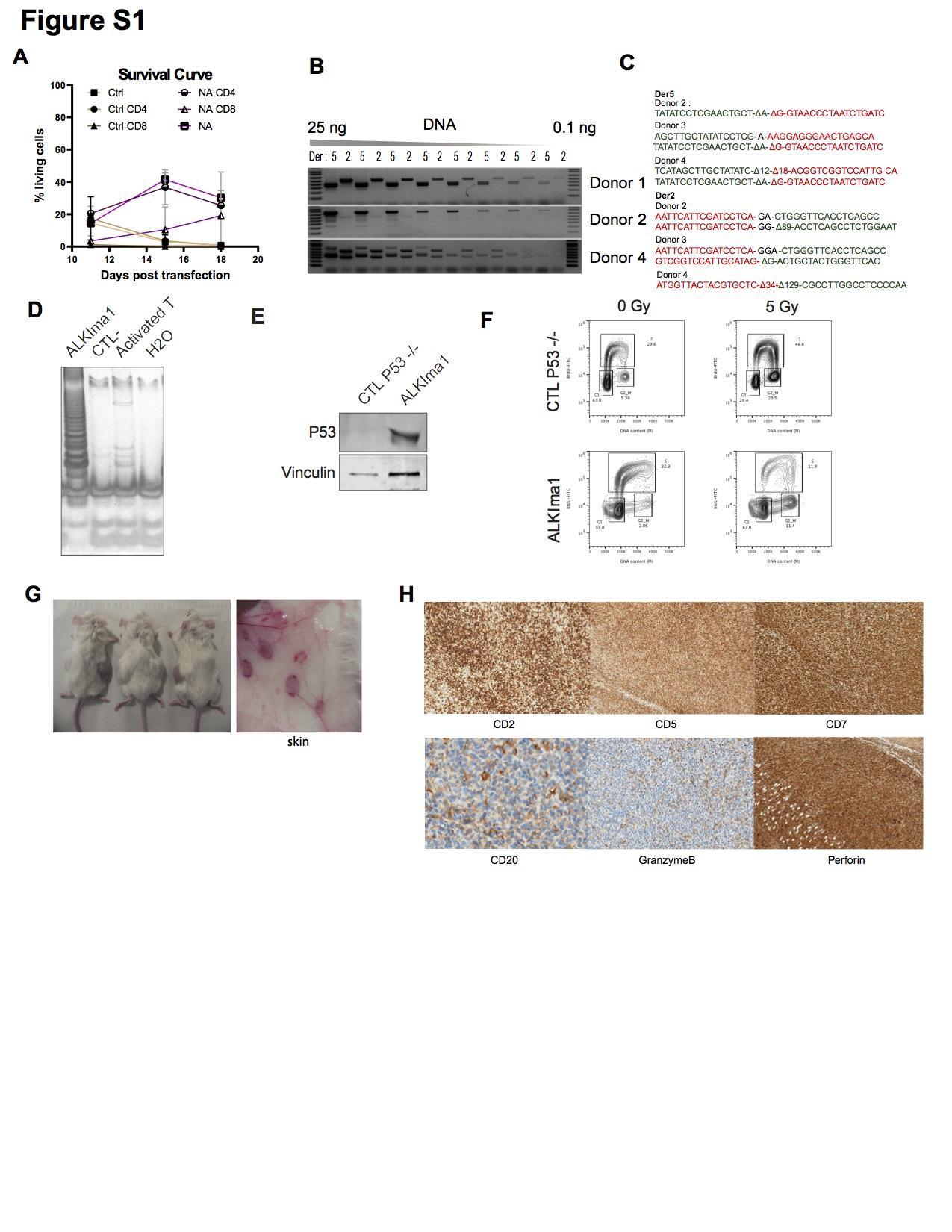


**
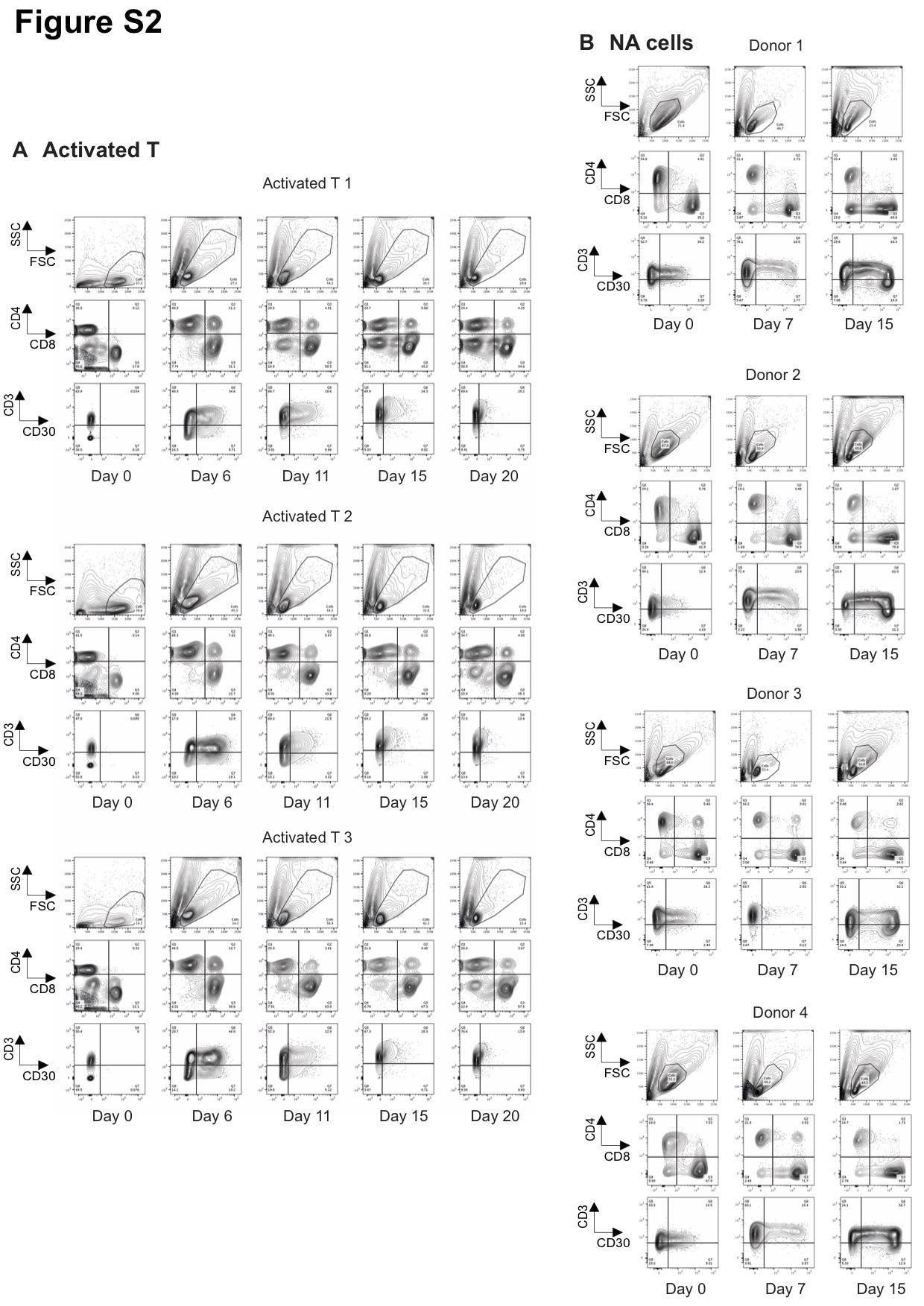
**

**
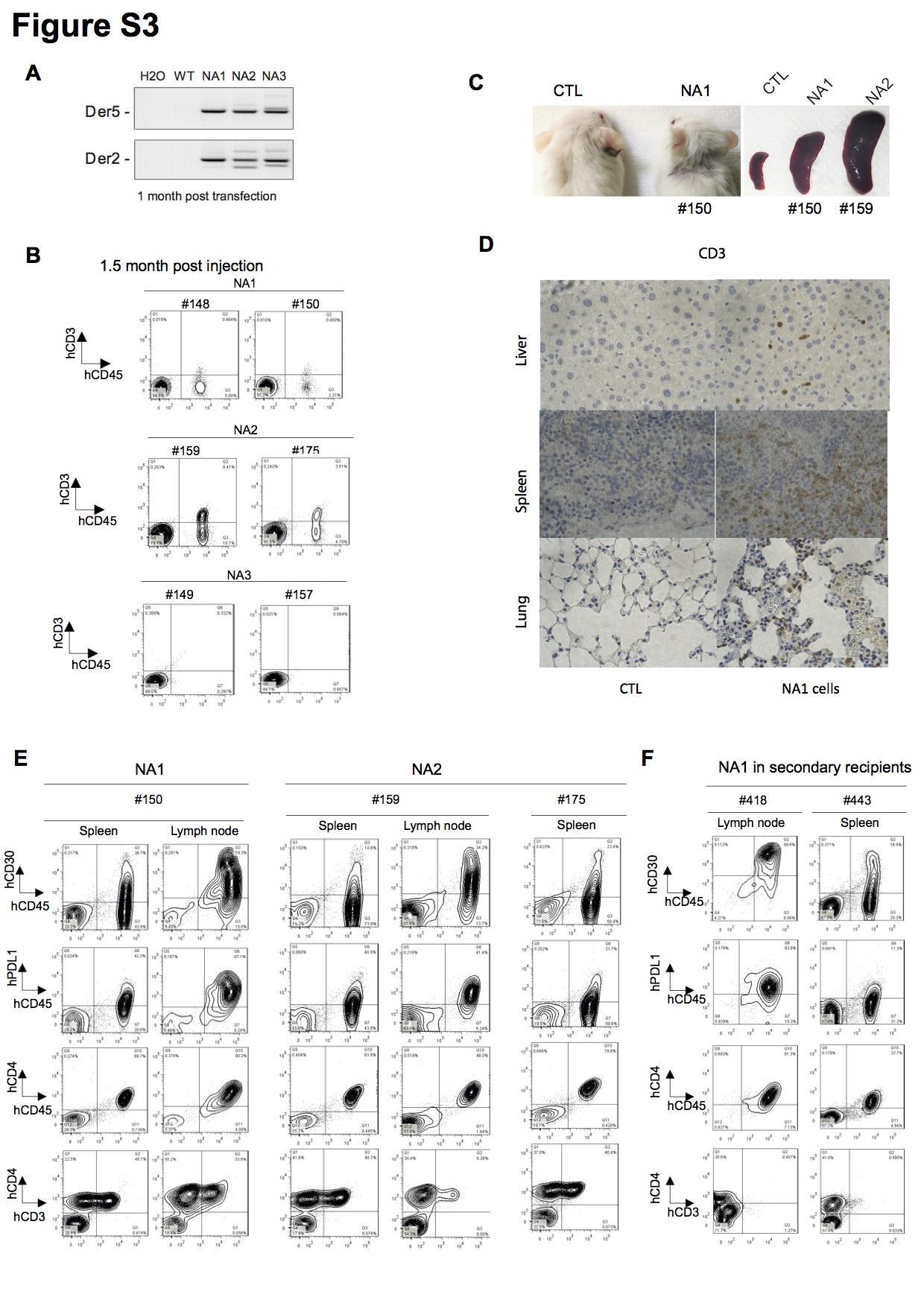
**

**
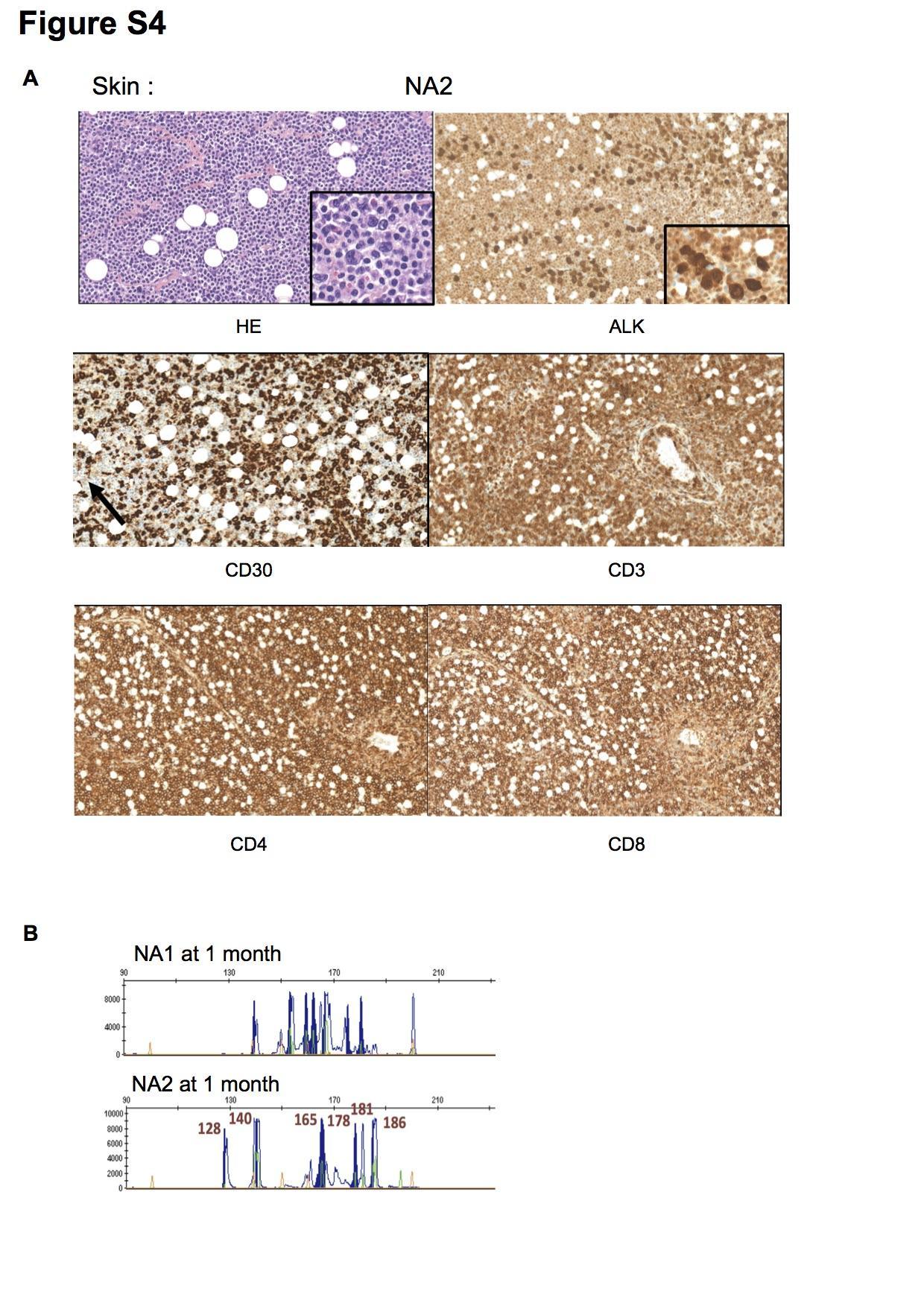
**

**
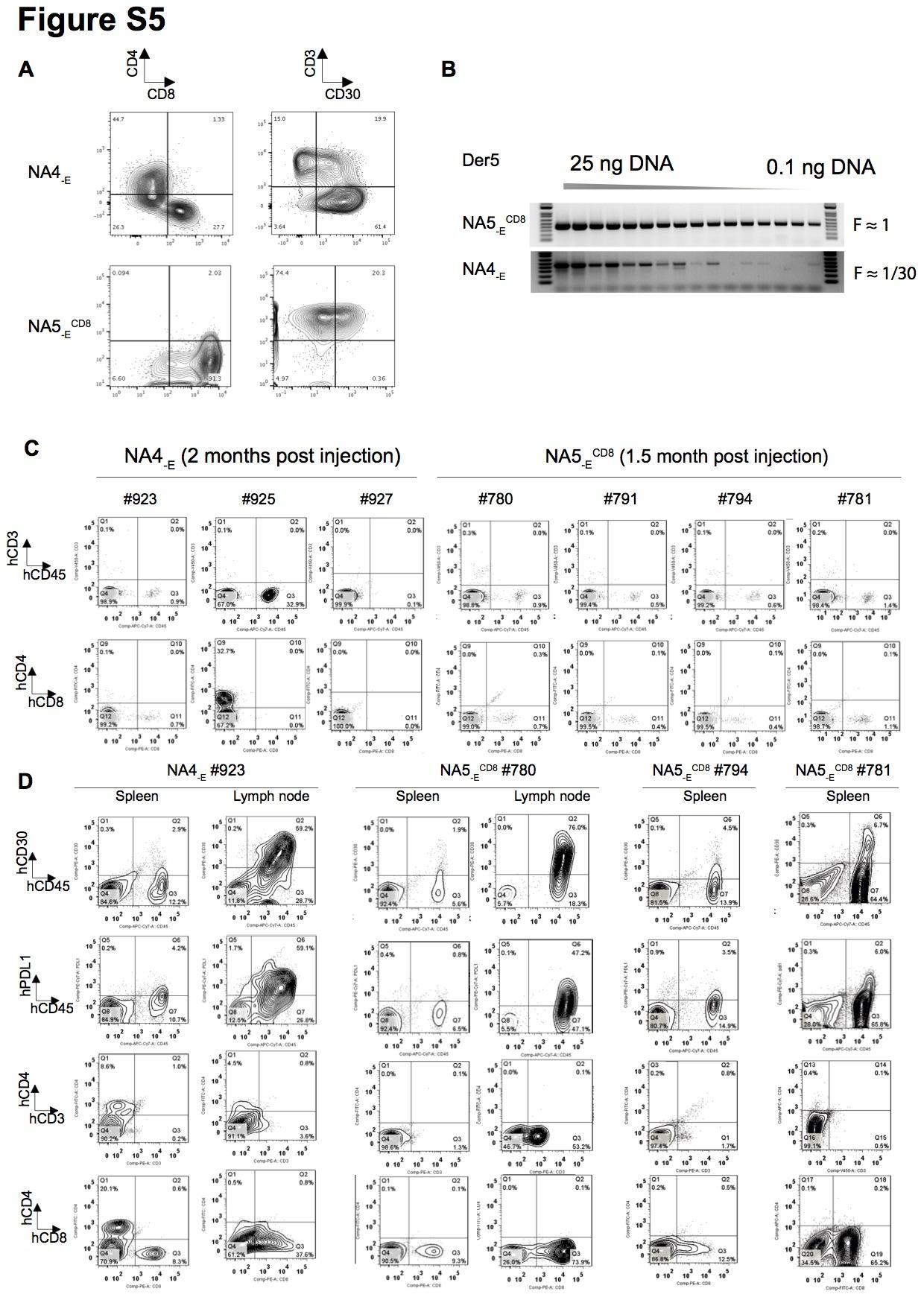
**

**
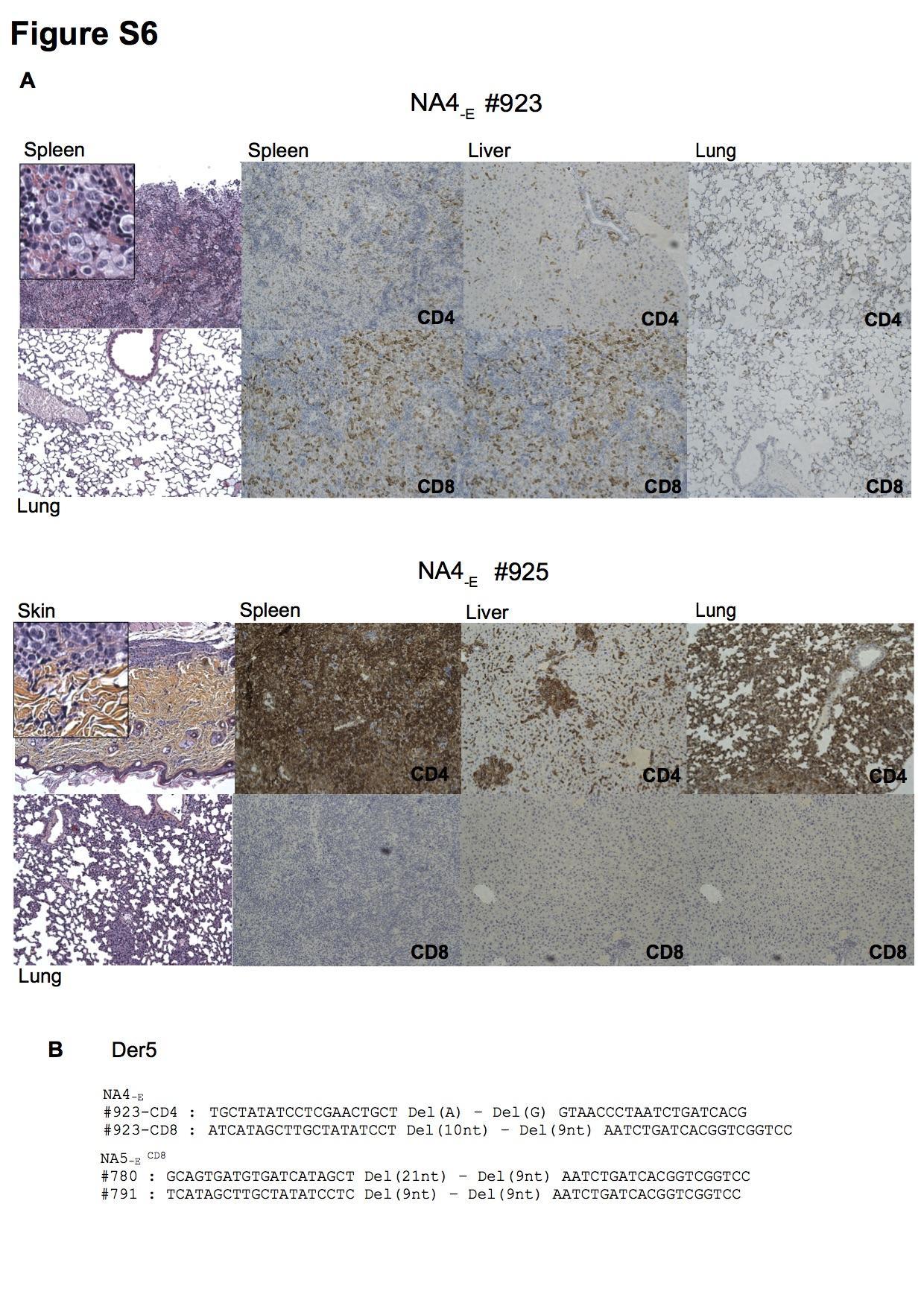
**

**
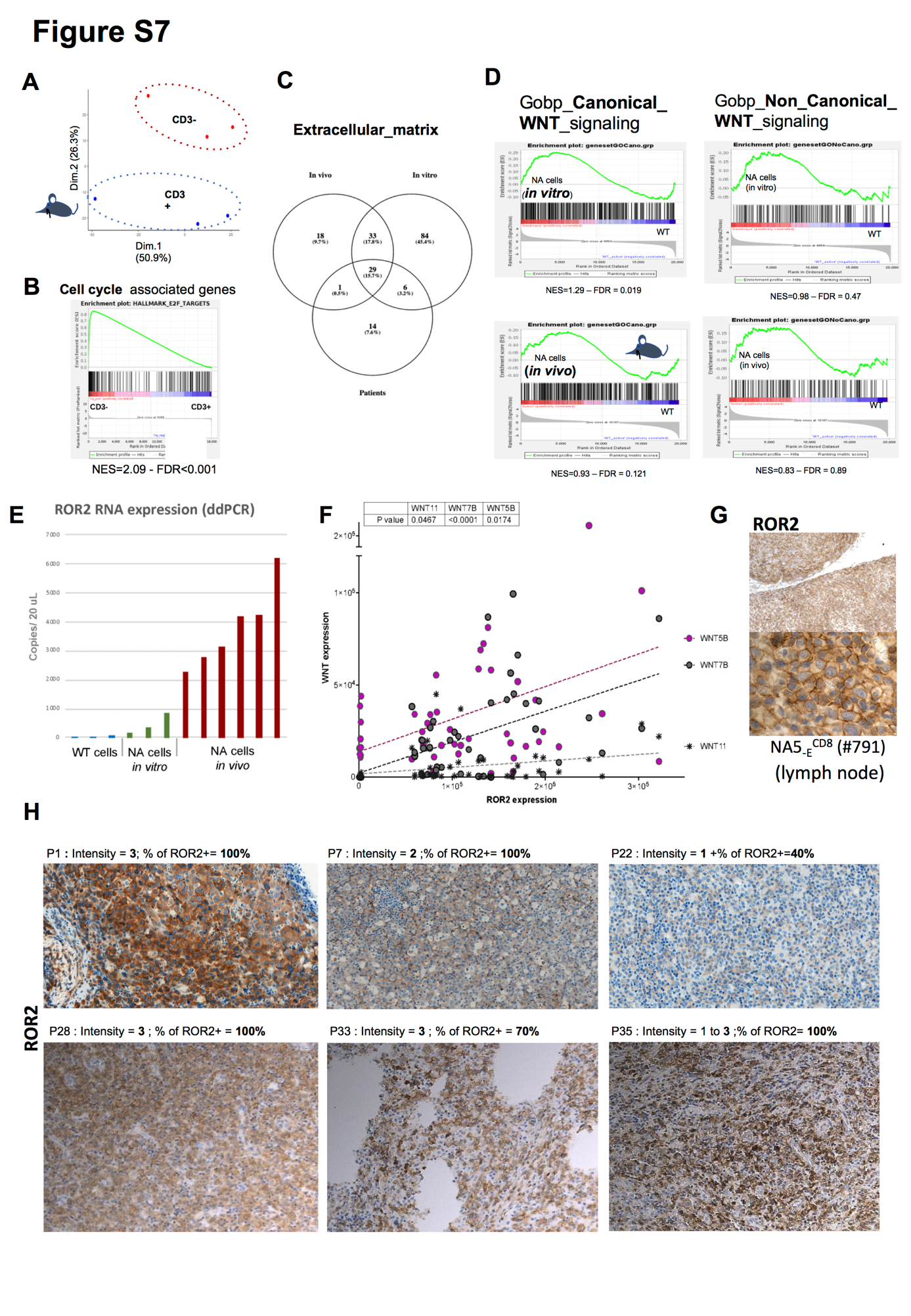
**

**
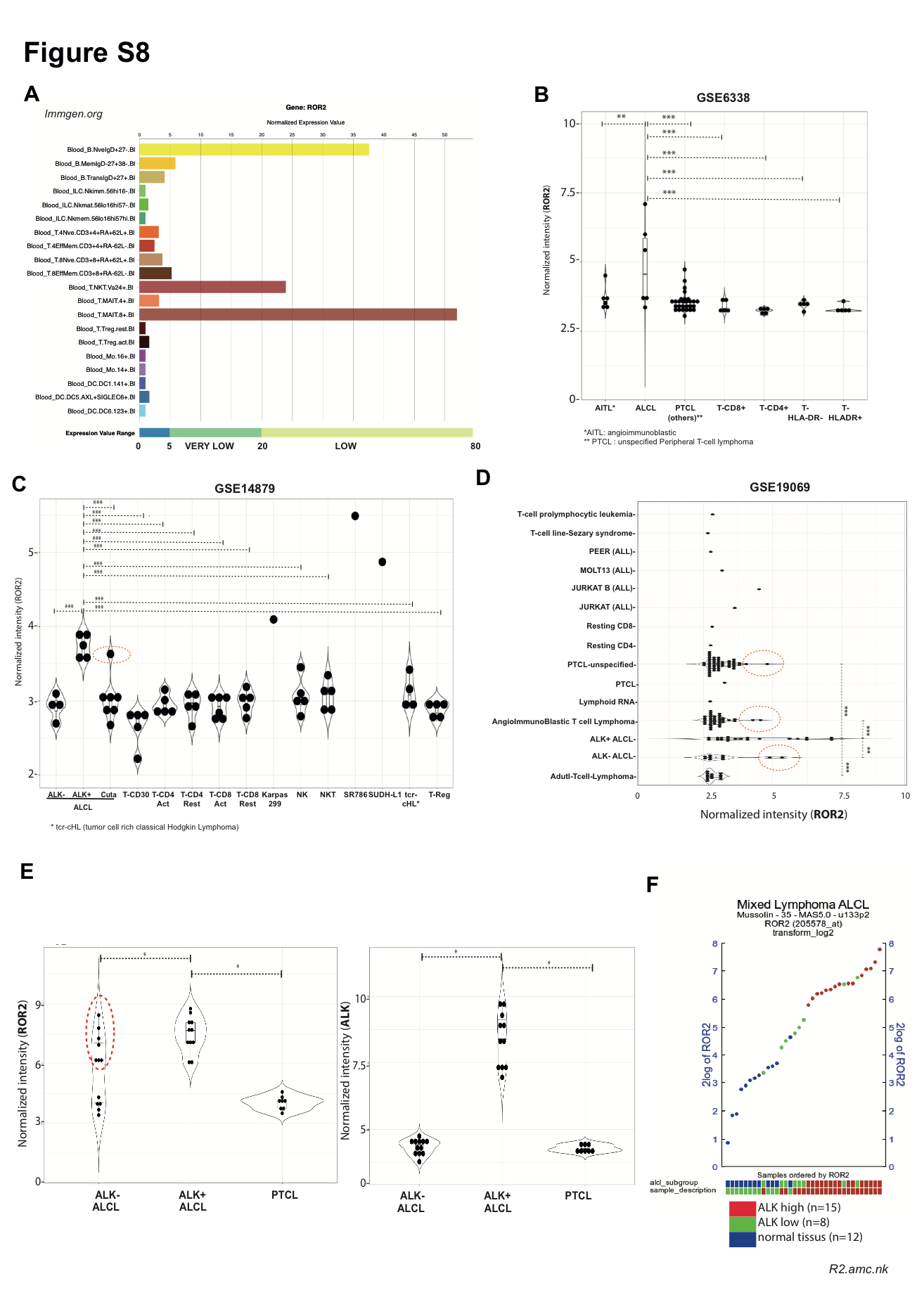
**
